# Supplementary material for: Ligand-based design of [18F]OXD-2314 for PET imaging in non-Alzheimer’s disease tauopathies
Source: Nat Commun. 2024 Jun 14;15:5109. doi: 10.1038/s41467-024-49258-1 (PMC11178805; doi:10.1038/s41467-024-49258-1)
Supplement: Supplementary file 1 — Supplementary Information [file 41467_2024_49258_MOESM1_ESM.pdf]

Supplementary Information

Ligand-based design of [^{18}F]OXD-2314 for PET imaging in non-Alzheimer's disease tauopathies

Anton Lindberg[1], Emily Murrell[1,2], Junchao Tong[1], N. Scott Mason[3], Daniel Sohn[4], Johan Sandell[5], Peter Ström[5], Jeffrey S. Stehouwer[3], Brian J. Lopresti[3], Jenny Viklund[4], Samuel Svensson[4,], Chester A. Mathis[3,*], and Neil Vasdev[1,2,*]*

[1]Azrieli Centre for Neuro-Radiochemistry, Brain Health Imaging Centre, Centre for Addiction and Mental Health, Toronto, Canada.

[2]Department of Psychiatry, University of Toronto, Canada

[3]Department of Radiology, University of Pittsburgh, USA.

[4]Oxiant Discovery, SE-15136 Södertälje, Sweden.

[5]Novandi Chemistry AB, SE-15136 Södertälje, Sweden

*Co-Corresponding authors : samuel.svensson@oxiantdiscovery.com; mathis@pitt.edu; neil.vasdev@utoronto.ca

Table of contents.

Supplementary Methods

In vitro characterization, radiosynthesis and rat PET imaging of [^{18}F]OXD-2188 and [^{18}F]OXD-2189 3

Chemistry methods 4

Supplementary Figures

In silico calculations for predicting BBB permeability of [^{18}F]OXD-2314 11

Human tissue sample information 12

Mouse pharmacology assay for OXD-2115 and OXD-2314 13

In vitro pharmacology screening 20

Radiometabolite analysis in rats 24

Dosimetry analysis in rats 26

PET scan and radiometabolites in NHP 28

Autoradiography using [^{3}H]OXD-2314 31

Representative homologous binding assay results of [^{3}H]OXD-2314 35

^{13}C- and ^{1}H-NMR spectra for compounds **1-8** including OXD-2314 37

Supplementary References 56

Supplementary Methods

In vitro characterization, radiosynthesis and rat PET imaging of [^{18}F]OXD-2188 and [^{18}F]OXD-2189

In vitro characterization. K_i values for OXD-21188 and OXD-2189 vs. [^{3}H]OXD-2115 in PSP tissue was 29.8 nM and 13.7nM respectively.

Radiochemistry. [^{18}F]OXD-2188 and [^{18}F]OXD-2189 were radiolabeled using the same methodology as for [^{18}F]OXD-2115.[1] [^{18}F]OXD-2188 and [^{18}F]OXD-2189 were synthesized in radiochemical purity above 95%, molar activity of 60.1 and 58.4 GBq/μmol, respectively, and radiochemical yield of 9% (not decay-corrected).

PET imaging. Three adult rats (two females and one male) were used in baseline PET imaging of [^{18}F]OXD-2188 and [^{18}F]OXD-2189 (16-23 MBq injected, with the male rat being scanned twice on two consecutive days). PET-CT imaging in normal rats showed initial uptake of only ~1.0 SUV for both [^{18}F]OXD-2188 (n=2) and [^{18}F]OXD-2189 (n=2) with rapid clearance of radioactivity from the brain (**Figure S1**).

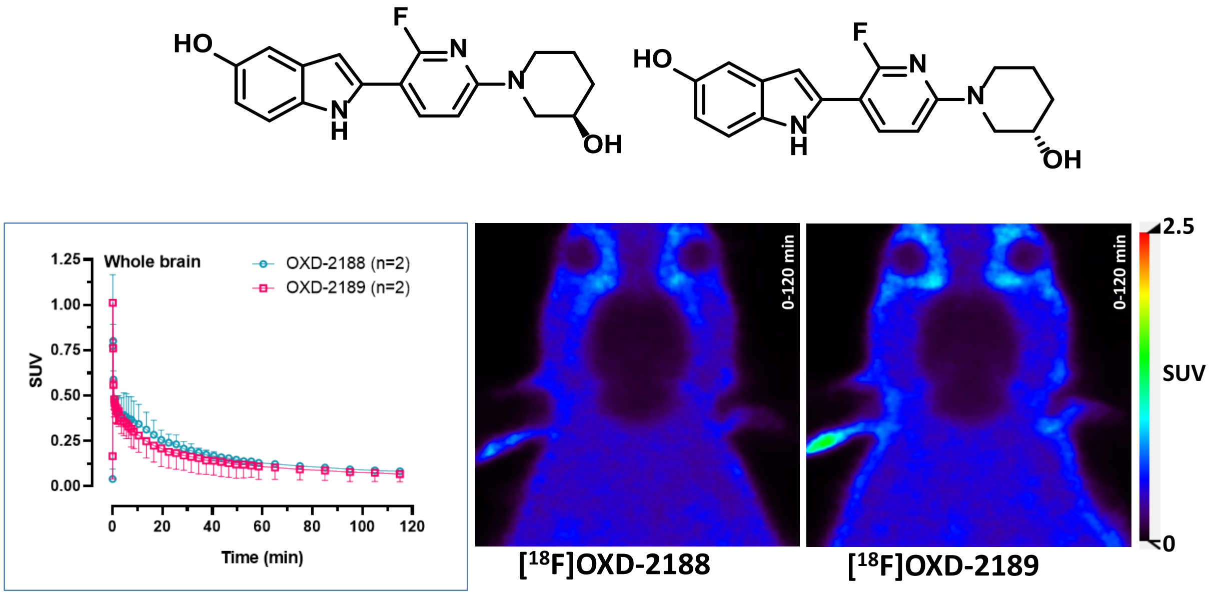

Figure S1. Structures of OXD-2188 and OXD-2189, time-activity curves and representative PET summation images from 0-120 min from PET-CT scans of [^{18}F]OXD-2188 and [^{18}F]OXD-2189 in rats.

Chemistry Methods.

All reagents and solvents used were analytical grade and commercially available. Anhydrous reactions were routinely used for reactions. Reactions were typically run under inert atmosphere of nitrogen (N_2). ^{1}H and ^{13}C NMR spectra were recorded on a Bruker Avance III 400MHz or an Agilent DD2 500MHz. High-resolution mass spectra (HRMS) were acquired using a JEOL AccuTOF Plus 4G with Direct Analysis in Real Time (DART) ion source. Microwave heating was performed in a Biotage Initiator 2.0. Flash column chromatography was performed using Silica gel 60 (0.040-0.063 mm) in a filter funnel or by using a Teledyne ISCO CombiFlash Rf with varying sizes (4 - 120 g) of Silicycle silica columns. TLC plates were Merck Silica gel 60 F254.

2-Fluoro-6-[(3*S*)-3-methoxypiperidin-1-yl]pyridine (**1**). (*S*)-3-Methoxypiperidine HCl (1.0 g, 6.6 mmol) was slurried in dioxane (8 mL) and 2,6-difluoropyridine (660 µL, 1.1 eq) was added followed by Hunig's base (3.0 ml, 2.6 eq). The reaction was heated to 100 °C for 5 h.

The cooled reaction mixture was taken into ethyl acetate, washed with water, treated with brine, dried using $MgSO_4$ and filtered. The solvent was removed *in vacuo*. The crude was purified by flash column chromatography (40 g silica, applied with hexane, eluted with 5-20% ethyl acetate/ hexane over 5 min) to give 2-fluoro-6-[(3*S*)-3-methoxypiperidin-1-yl]pyridine (1.03 g, 75% yield, TLC 10% ethyl acetate/ hexane Rf 0.14). ^{1}H NMR (500 MHz, dmso-d6) δ 7.59 (ddd, *J* = 9.2, 8.3, 7.7 Hz, 1H), 6.69 – 6.60 (m, 1H), 6.16 (ddd, *J* = 7.7, 2.9, 0.4 Hz, 1H), 3.86 (dq, *J* = 11.0, 1.3 Hz, 1H), 3.64 (dddt, *J* = 13.2, 5.6, 3.9, 0.9 Hz, 1H), 3.25 (s, 3H), 3.24 – 3.15 (m, 3H), 1.95 – 1.84 (m, 1H), 1.68 (dddd, *J* = 13.3, 7.2, 6.0, 3.5 Hz, 1H), 1.52 – 1.43 (m, 1H), 1.39 (dtt, *J* = 13.2, 9.2, 3.7 Hz, 1H). ^{13}C NMR (126 MHz, dmso-d6) δ 163.41, 161.56, 158.28, 158.15, 142.90, 142.83, 103.56, 103.52, 94.90, 94.61, 74.64, 55.93, 48.37, 45.00, 29.95, 22.09. HRMS (DART+) *m/z* calculated for 211.12482, found 211.12447

3-Bromo-2-fluoro-6-[(3*S*)-3-methoxypiperidin-1-yl]pyridine (**2**). 2-Fluoro-6-[(3*S*)-3-methoxypiperidin-1-yl]pyridine (1.03 g, 4.9 mmol) was dissolved in acetonitrile (20 mL), cooled on an ice-bath and NBS (0.87 g, 1 eq) was added in 2 portions. The reaction was allowed to stir at 0 °C for 5 min then stirred at rt for 30 min. Diethylether was added to the mixture, the solution was washed with water, treated with brine, dried using $MgSO_4$ and filtered. The solvent was removed *in vacuo*. The crude was purified by flash column chromatography (40 g silica, applied with hexane/ DCM, eluted with 5-20% ethyl acetate/ hexane over 5 min) to give 3-bromo-2-fluoro-6-[(3*S*)-3-methoxypiperidin-1-yl]pyridine (1.35 g, 96% yield, TLC 20% ethyl acetate/ hexane Rf 0.19). ^{1}H NMR (500 MHz, dmso-d6) δ 7.75 (dd, *J* = 9.6, 8.7 Hz, 1H), 6.67 (dd, *J* = 8.8, 1.9 Hz, 1H), 3.78 (ddt, *J* = 12.9, 3.2, 1.1 Hz, 1H), 3.57 (ddd, *J* = 13.3, 6.7, 3.8 Hz, 1H), 3.33 – 3.25 (m, 2H), 3.24 – 3.19 (m, 1H), 1.87 (dqd, *J* = 11.3, 3.8, 1.9 Hz, 1H), 1.72 – 1.61 (m, 1H), 1.54 – 1.46 (m, 1H), 1.43 – 1.34 (m, 1H). ^{13}C NMR (126 MHz, dmso-d6) δ 158.61, 157.07, 156.95, 156.78, 144.96, 144.94, 106.04, 106.01, 85.85, 85.54, 74.51, 55.98, 48.40, 45.16, 29.70, 21.85. HRMS (DART+) *m/z* calculated for 289.03349, found 289.03367

t-Butyl 5-[(t-butyldimethylsilyl)oxy]-2-{2-fluoro-6-[(3*S*)-3-methoxypiperidin-1-yl]pyridin-3-yl}-1*H*-indole-1-carboxylate (**3**). 3-Bromo-2-fluoro-6-[(3*S*)-3-methoxypiperidin-1-yl]pyridine (580 mg, 2 mmol) and {1-[(t-butoxy)carbonyl]-5-[(t-butyldimethylsilyl)oxy]-1*H*-indol-2-yl}boronic acid (1.1 g, 1.4 eq) were dissolved in dioxane (12 mL) in a 20 mL microwave vial (Biotage). The solution was bubbled with N_2 for 5 min, then Pd(dppf)Cl_2 DCM (82 mg, 5 mol%) was added followed by 2 M K_2CO_3 (3 mL, 3 eq). The solution was again bubbled with N_2 for 5 min, capped then put in a pre-heated oil bath. The reaction was run at 90 °C for 1 h. The aqueous was removed from the cooled reaction then was diluted with ethyl acetate, dried using $MgSO_4$ and filtered. The solvent was removed *in vacuo*. The purification was done by flash column

chromatography (40 g silica, applied with DCM, eluted with 5-15% ethyl acetate/ hexane over 6 min) to give t-butyl 5-[(t-butyldimethylsilyl)oxy]-2-{2-fluoro-6-[(3*S*)-3-methoxypiperidin-1-yl]pyridin-3-yl}-1*H*-indole-1-carboxylate (1.17 g, 98% yield, TLC 20% ethyl acetate/ hexane Rf 0.35). ^{1}H NMR (500 MHz, dmso-d6) δ 7.92 (dt, *J* = 8.9, 0.7 Hz, 1H), 7.66 (dd, *J* = 10.2, 8.3 Hz, 1H), 7.01 (dd, *J* = 2.4, 0.5 Hz, 1H), 6.83 (dd, *J* = 8.9, 2.5 Hz, 1H), 6.76 (dd, *J* = 8.4, 2.1 Hz, 1H), 6.59 (d, *J* = 0.7 Hz, 1H), 3.89 (dd, *J* = 13.1, 3.3 Hz, 1H), 3.67 (ddd, *J* = 13.2, 6.4, 3.8 Hz, 1H), 3.62 – 3.52 (m, 4H), 3.39 – 3.31 (m, 2H), 3.27 (s, 3H), 3.24 (tt, *J* = 7.4, 3.4 Hz, 1H), 1.92 (ddt, *J* = 11.4, 7.4, 3.8 Hz, 1H), 1.78 – 1.65 (m, 5H), 1.53 (dtd, *J* = 12.5, 8.3, 3.9 Hz, 1H), 1.45 – 1.36 (m, 1H), 1.34 (s, 9H), 0.95 (s, 9H). ^{13}C NMR (126 MHz, dmso-d6) δ 150.88, 149.35, 142.06, 133.99, 131.56, 129.63, 117.46, 115.44, 110.20, 109.68, 102.72, 102.44, 102.19, 83.27, 74.16, 67.01, 55.57, 48.08, 44.84, 29.53, 27.18, 25.63, 25.12, 21.57, 17.98, -4.53. HRMS (DART+) *m/z* calculated for 556.29880, found 556.29868

t-Butyl 2-{2-fluoro-6-[(3*S*)-3-methoxypiperidin-1-yl]pyridin-3-yl}-5-hydroxy-1*H*-indole-1-carboxylate (4). t-Butyl 5-[(t-butyldimethylsilyl)oxy]-2-{2-fluoro-6-[(3*S*)-3-methoxypiperidin-1-yl]pyridin-3-yl}-1*H*-indole-1-carboxylate (1.1 g, 2.0 mmol) was dissolved in THF (20 mL), cooled on an ice-bath and a 1 M tetrabutylammonium fluoride solution (2.2 mL, 1.1 eq) was added. The reaction stirred 10 min at 0 °C. The reaction was diluted with ethyl acetate, treated with brine, dried $MgSO_4$ and filtered. The solvent was removed *in vacuo*. The purification was done by flash column chromatography (40 g silica, applied with DCM, eluted with 30-45% ethyl acetate/ hexane over 5 min) to give t-butyl 2-{2-fluoro-6-[(3*S*)-3-methoxypiperidin-1-yl]pyridin-3-yl}-5-hydroxy-1*H*-indole-1-carboxylate (0.84 g, 98% yield, TLC Rf 45% ethyl acetate/ hexane Rf 0.28). ^{1}H NMR (500 MHz, dmso-d6) δ 9.18 (s, 1H), 7.87 (dt, *J* = 8.8, 0.7 Hz, 1H), 7.63 (dd, *J* = 10.2, 8.3 Hz, 1H), 6.89 (dd, *J* = 2.5, 0.5 Hz, 1H), 6.77 (dd, *J* = 8.9, 2.5 Hz, 1H), 6.72 (dd, *J* = 8.4, 2.1 Hz, 1H), 6.53

(d, J = 0.7 Hz, 1H), 3.88 (dd, J = 13.1, 3.3 Hz, 1H), 3.65 (ddd, J = 13.2, 6.3, 3.8 Hz, 1H), 3.36 – 3.28 (m, 3H), 3.22 (tt, J = 7.4, 3.4 Hz, 1H), 1.94 – 1.85 (m, 1H), 1.69 (dtt, J = 13.4, 6.9, 3.5 Hz, 1H), 1.52 (tdd, J = 12.5, 7.7, 3.9 Hz, 1H), 1.45 – 1.35 (m, 1H), 1.33 (s, 8H). ^{13}C NMR (126 MHz, dmso-d6) δ 170.31, 159.27, 157.41, 157.17, 157.04, 153.33, 149.45, 142.00, 141.97, 133.62, 133.58, 130.25, 129.71, 115.39, 113.35, 109.69, 104.99, 102.73, 102.67, 102.64, 102.49, 83.11, 82.96, 74.17, 59.75, 55.56, 48.11, 44.85, 30.96, 29.53, 27.20, 22.07, 21.57, 20.74, 20.04, 14.07, 13.95, 13.92. HRMS (DART+) *m/z* calculated for 442.21252, found 442.21266

2-{2-Fluoro-6-[(3*S*)-3-methoxypiperidin-1-yl]pyridin-3-yl}-1*H*-indol-5-ol (OXD-2314) In a 20mL microwave vial, t-butyl 2-{2-fluoro-6-[(3*S*)-3-methoxypiperidin-1-yl]pyridin-3-yl}-5-hydroxy-1*H*-indole-1-carboxylate (0.84 g, 1.9 mml) was dissolved in methanol (15 mL). The reaction was subjected to the microwave for 45 min at 150 °C. The solvent was removed, the remains were stirred with DCM and the solid was filtered to give 2-{2-fluoro-6-[(3*S*)-3-methoxypiperidin-1-yl]pyridin-3-yl}-1*H*-indol-5-ol (300 mg, 47% yield, TLC Rf 40% ethyl acetate/ hexane Rf 0.15). ^{1}H NMR (400 MHz, dmso-d6) δ 11.00 (s, 1H), 8.69 (s, 1H), 8.12 (dd, J = 10.7, 8.6 Hz, 1H), 7.23 (dt, J = 8.6, 0.7 Hz, 1H), 6.91 (dd, J = 8.6, 2.1 Hz, 1H), 6.87 (d, J = 2.3 Hz, 1H), 6.65 (dd, J = 8.6, 2.3 Hz, 1H), 6.60 – 6.53 (m, 1H), 3.97 (d, J = 12.1 Hz, 1H), 3.75 (ddd, J = 13.0, 6.3, 3.7 Hz, 1H), 3.36 (s, 7H), 2.08 – 1.91 (m, 1H), 1.79 (ddt, J = 13.4, 6.8, 3.4 Hz, 1H), 1.67 – 1.42 (m, 2H). ^{13}C NMR (126 MHz, dmso-d6) δ 159.19, 157.31, 156.35, 156.22, 151.28, 139.53, 132.14, 132.08, 131.47, 129.80, 111.96, 111.78, 104.28, 103.94, 101.65, 101.42, 99.69, 74.65, 56.00, 48.43, 45.10, 29.87, 22.08. ^{19}F NMR (377 MHz, DMSO) δ -67.79, -67.82). HRMS (DART+) *m/z* calculated for 342.16123, found 342.16119

2-[(3*S*)-3-Methoxypiperidin-1-yl]-6-nitropyridine (**5**). 2-Chloro-6-nitropyridine (0.63 g, 4 mmol) and (*S*)-3-methoxypiperidine HCl (0.9 g, 1.5 eq) were slurried in dioxane (6 mL) followed

by the addition of Hunig's base (2.9 mL, 1.5 eq). The reaction was heated to 100 °C for 19 h. The cooled reaction mixture was taken into ethyl acetate, washed with water, treated with brine, dried using $MgSO_4$ and filtered. The solvent was removed *in vacuo*. The crude was purified by flash column chromatography (40 g silica, applied with hexane, eluted with 15-30% ethyl acetate/ hexane over 8 min) to give 2-[(3*S*)-3-methoxypiperidin-1-yl]-6-nitropyridine (0.63 g, TLC 30% ethyl acetate/ hexane Rf 0.22). ^{1}H NMR (500 MHz, dmso-d6) δ 7.78 (dd, *J* = 8.6, 7.5 Hz, 1H), 7.34 (d, *J* = 7.5 Hz, 1H), 7.25 (d, *J* = 8.6 Hz, 1H), 3.86 (ddt, *J* = 13.4, 3.4, 1.0 Hz, 1H), 3.66 (ddd, *J* = 13.3, 6.9, 3.7 Hz, 1H), 3.50 – 3.41 (m, 2H), 3.30 – 3.26 (m, 1H), 3.25 (s, 3H), 1.92 – 1.84 (m, 1H), 1.70 (dtd, *J* = 17.0, 7.3, 3.7 Hz, 1H), 1.58 – 1.49 (m, 1H), 1.45 – 1.36 (m, 1H). ^{13}C NMR (126 MHz, dmso-d6) δ 157.77, 155.75, 141.11, 113.09, 104.84, 74.72, 74.57, 56.67, 55.95, 55.33, 48.07, 44.96, 29.71, 21.88. HRMS (DART+) *m/z* calculated for 238.11862, found 238.11834

3-Bromo-6-[(3*S*)-3-methoxypiperidin-1-yl]-2-nitropyridine (**6**). 2-[(3*S*)-3-Methoxypiperidin-1-yl]-6-nitropyridine (0.61 g, 2.6 mmol) was dissolved in acetonitrile (15 mL), cooled on an ice-bath and NBS (0.65 g, 1 eq) was added. The reaction was allowed to stir at 0 °C for 5 min then stirred at rt for 60 min. The reaction was taken into ethyl acetate, treated with brine, treated with brine, dried using $MgSO_4$ and filtered. The solvent was removed *in vacuo*. The crude was purified by flash column chromatography (40 g silica, applied with hexane/ DCM, eluted with 15-30% ethyl acetate/ hexane over 6 min) to give 3-bromo-6-[(3*S*)-3-methoxypiperidin-1-yl]-2-nitropyridine (0.74 g, TLC 60% ethyl acetate/ hexane Rf 0.30). ^{1}H NMR (400 MHz, $CDCl_3$) δ 7.70 (d, *J* = 9.0 Hz, 1H), 6.72 (d, *J* = 9.0 Hz, 1H), 3.85 (ddt, *J* = 13.2, 3.4, 1.1 Hz, 1H), 3.68 (ddd, *J* = 13.3, 6.6, 3.8 Hz, 1H), 3.52 – 3.31 (m, 6H), 2.05 – 1.94 (m, 1H), 1.88 (ddp, *J* = 14.2, 7.3, 3.6 Hz, 1H), 1.69 (ddp, *J* = 12.8, 8.7, 3.9 Hz, 1H), 1.61 – 1.48 (m, 1H). ^{13}C NMR (126 MHz, $CDCl_3$) δ

156.28, 144.12, 110.93, 93.14, 74.58, 56.40, 48.46, 45.43, 29.58, 21.80. HRMS (DART+) *m/z* calculated for 316.02913, found 316.02855

t-Butyl 5-[(t-butyldimethylsilyl)oxy]-2-{6-[(3*S*)-3-methoxypiperidin-1-yl]-2-nitropyridin-3-yl}-1*H*-indole-1-carboxylate (**7**). 3-Bromo-6-[(3*S*)-3-methoxypiperidin-1-yl]-2-nitropyridine (316 mg, 1 mmol) and {1-[(t-butoxy)carbonyl]-5-[(t-butyldimethylsilyl)oxy]-1*H*-indol-2-yl}boronic acid (550 mg, 1.4 eq) were dissolved in dioxane (6 mL) in a 20 ml microwave vial (Biotage). The solution was bubbled with N_2 for 2 min, then Pd(dppf)Cl_2 DCM (41 mg, 5 mol%) was added followed by 2 M K_2CO_3 (31.5mL, 3 eq). The solution was again bubbled with N_2 for 5 min, capped then put in a pre-heated oil bath. The reaction was run at 90 °C for 1 h. The aqueous was removed from the cooled reaction then was diluted with ethyl acetate, dried using $MgSO_4$ and filtered. The solvent was removed *in vacuo*. The purification was performed by flash column chromatography (40 g silica, applied with DCM, eluted with 10-20% ethyl acetate/ hexane over 6 min) to give t-butyl 5-[(t-butyldimethylsilyl)oxy]-2-{6-[(3*S*)-3-methoxypiperidin-1-yl]-2-nitropyridin-3-yl}-1*H*-indole-1-carboxylate (334 mg, 57% yield, TLC 20% ethyl acetate/ hexane Rf 0.16). ^{1}H NMR (500 MHz, dmso-d6) δ 7.97 (dt, *J* = 9.0, 0.7 Hz, 1H), 7.77 (d, *J* = 8.7 Hz, 1H), 7.27 (d, *J* = 8.7 Hz, 1H), 7.04 – 6.99 (m, 1H), 6.85 (dd, *J* = 9.0, 2.5 Hz, 1H), 6.57 (d, *J* = 0.7 Hz, 1H), 3.85 (dd, *J* = 13.5, 3.1 Hz, 1H), 3.66 (ddd, *J* = 13.0, 7.0, 3.7 Hz, 1H), 3.58 (dd, *J* = 13.3, 6.9 Hz, 2H), 3.29 (td, *J* = 6.6, 3.1 Hz, 1H), 3.27 (s, 3H), 1.90 (ddt, *J* = 12.3, 8.0, 3.9 Hz, 1H), 1.76 – 1.67 (m, 1H), 1.60 (ddd, *J* = 12.1, 9.8, 5.8 Hz, 1H), 1.48 – 1.37 (m, 1H), 1.28 (s, 12H), 0.95 (s, 12H), 0.18 (s, 6H). ^{13}C NMR (126 MHz, dmso-d6) δ 157.00, 154.26, 151.41, 149.57, 143.55, 134.78, 131.73, 130.17, 118.18, 116.45, 111.10, 110.83, 110.76, 110.46, 83.81, 74.56, 56.04, 48.16, 45.19, 29.72, 27.60, 26.08, 21.82, 18.42, -4.08. HRMS (DART+) *m/z* calculated for 583.29598, found 583.29561

t-Butyl 5-hydroxy-2-{6-[(3*S*)-3-methoxypiperidin-1-yl]-2-nitropyridin-3-yl}-1*H*-indole-1-carboxylate (**8**). t-Butyl 5-[(t-butyldimethylsilyl)oxy]-2-{6-[(3*S*)-3-methoxypiperidin-1-yl]-2-nitropyridin-3-yl}-1*H*-indole-1-carboxylate (236 g, 0.40 mmol) was dissolved in THF (7 mL), cooled on an ice-bath and a 1 M TBAF solution (450 µL, 1.1 eq) was added. The reaction stirred 10 min at 0 °C. The reaction was diluted with ethyl acetate, treated with brine, dried $MgSO_4$ and filtered. The solvent was removed *in vacuo*. The purification was performed by flash column chromatography (12 g silica, applied with DCM, eluted with 25-40% ethyl acetate/ hexane over 5 min) to give t-butyl 5-hydroxy-2-{6-[(3*S*)-3-methoxypiperidin-1-yl]-2-nitropyridin-3-yl}-1*H*-indole-1-carboxylate (142 mg, 75% yield, TLC Rf 40% ethyl acetate/ hexane Rf 0.16). ^{1}H NMR (400 MHz, $CDCl_3$) δ 8.03 (d, J = 8.9 Hz, 1H), 7.56 (d, J = 8.6 Hz, 1H), 6.91 (d, J = 2.5 Hz, 1H), 6.88 – 6.81 (m, 2H), 6.35 (d, J = 0.7 Hz, 1H), 3.95 (dd, J = 13.3, 3.3 Hz, 1H), 3.76 (ddd, J = 13.4, 6.5, 3.8 Hz, 1H), 3.56 (dd, J = 13.3, 7.2 Hz, 1H), 3.42 (s, 4H), 3.37 (tt, J = 7.2, 3.5 Hz, 1H), 2.00 (dq, J = 11.6, 3.6 Hz, 1H), 1.88 (dtt, J = 13.9, 7.1, 3.6 Hz, 1H), 1.69 (dtd, J = 12.5, 8.2, 3.7 Hz, 1H), 1.55 (ddt, J = 15.2, 8.8, 4.1 Hz, 1H), 1.39 (s, 9H). ^{13}C NMR (101 MHz, $CDCl_3$) δ 156.82, 151.78, 149.82, 142.77, 134.69, 131.55, 130.02, 116.72, 113.52, 111.46, 110.10, 109.35, 105.52, 83.70, 77.36, 77.04, 76.72, 74.81, 56.41, 48.38, 45.46, 29.85, 27.80, 21.99. HRMS (DART+) *m/z* calculated for 469.20816, found 469.20799

Supplementary Figures

In silico calculations for predicting BBB permeability of [^{18}F]OXD-2314.

OXD-2115

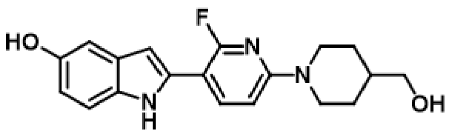

CNS MPO and PET MPO			
Property	Value	CNS MPO	CNS PET MPO
ClogP	2.35	1.000	1.000
ClogD	2.99	0.505	0.000
TPSA	68.09	1.000	0.791
MW	341	1.000	0.121
HBD	3	0.167	0.000
pKa	14	0.000	0.000
	Total Score	3.7	1.9

BBB Score Calculator		
Property	Value	T_0
Number of Aromatic Rings (Aro_R)	3	0.69
Number of Heavy Atoms (HA)	25	0.92
Molecular Weight (MW)	341.00	
Number of Hydrogen Bond Acceptor (HBA)	5	
Number of Hydrogen Bond Donor (HBD)	3	
MWHBN [MWHBN = (MW^(-0.5)*HBN), where HBN=HBA+HBD]	0.43	0.35
Topological Polar Surface Area(TPSA)	68.09	0.52
pKa	14	0.00
BBB SCORE		3.18

OXD-2314

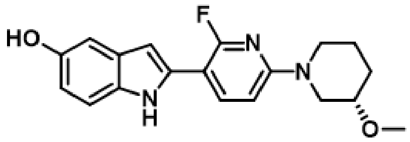

CNS MPO and PET MPO			
Property	Value	CNS MPO	CNS PET MPO
ClogP	3.35	0.825	0.542
ClogD	3.11	0.445	0.000
TPSA	57.09	1.000	1.000
MW	341	1.000	0.121
HBD	2	0.500	0.000
pKa	14	0.000	0.000
	Total Score	3.8	1.7

BBB Score Calculator		
Property	Value	T_0
Number of Aromatic Rings (Aro_R)	3	0.69
Number of Heavy Atoms (HA)	25	0.92
Molecular Weight (MW)	341.00	
Number of Hydrogen Bond Acceptor (HBA)	5	
Number of Hydrogen Bond Donor (HBD)	2	
MWHBN [MWHBN = (MW^(-0.5)*HBN), where HBN=HBA+HBD]	0.38	0.56
Topological Polar Surface Area(TPSA)	57.09	0.60
pKa	14	0.00
BBB SCORE		3.65

Figure S2. *In silico* evaluation of CBD-2115 and OXD-2314 using CNS MPO, CNS PET MPO and BBB score. Chemdraw Professional 15.0 was used for all physicochemical properties except $logD_{7.4}$ which was obtained experimentally.

Human Tissue sample information

Table S1. Summary of demographic and neuropathologic characteristics of donors and postmortem brain tissues. Key to scores: 1 = mild, 2 = moderate, 3 = severe ; or 1 = small, 2 = moderate, or 3 = large numbers of given hallmark.

Case	Sex	Age	Clinical Diagnosis	Neuropath Diagnosis	PMI (hrs)	Frozen Block Anatomical Location	Abeta_ CERAD Neuritic plaques	Abeta Diffuse Plaques	Abeta CAA	CP13 Neurofibrillary tangles	CP13 Pick bodies	CP13 Neuronal cytoplasmic inclusions	CP13 Globose tangles	CP13 Astrocytic plaques	CP13 Tufted astrocytes	CP13 Thorny astrocytes	CP13 Neuropil threads /grains	CP13 White matter threads /grains	CP13 Glial cytoplasmic inclusions	aSyn Lewy Bodies	aSyn Lewy Neurites	aSyn Glial cytoplasmic inclusions
AD1	M	63	AD	AD	6.0	Middle/inferior frontal gyrus, anterior	3	3	0	3	0	0	0	0	0	0	3	1	0	0	0	0
AD2	M	64	AD	AD	6.8	Middle/inferior frontal gyrus, anterior	3	3	1	3	0	0	0	0	0	0	3	1	0	0	0	0
AD3	F	66	AD	AD	15.4	Middle/inferior frontal gyrus, anterior	3	3	2	3	0	0	0	0	0	0	3	1	0	0	0	0
PSP1	M	63	PSP	PSP	4.4	Superior frontal gyrus	0	0	0	0	0	3	0	0	3	1	2	2	3	0	0	0
PSP2	F	70	PSP	PSP	7.2	Superior frontal gyrus	0	0	0	0	0	2	0	0	3	1	1	1	3	0	0	0
PSP3	M	68	PSP	PSP	10.1	Superior frontal gyrus	0	0	0	0	0	2	1	0	3	1	1	0	2	0	0	0
PSP4	F	66	PSP	PSP	9.2	Putamen & Globus Pallidus	0	0	0	0	0	2	1	0	3	0	1	1	3	0	0	0
CBD1	M	68	PNFA	CBD	7.4	Middle/inferior frontal gyrus, anterior	0	0	0	0	0	3	0	3	0	0	3	3	2	0	0	0
CBD2	F	70	CBS	CBD	7.4	Middle/inferior frontal gyrus, anterior	0	0	0	0	0	3	0	3	0	1	3	3	3	0	0	0
CBD3	F	63	CBS	CBD	5.0	Middle/inferior frontal gyrus, anterior	0	0	0	0	0	2	0	3	0	0	2	2	1	0	0	0
CBD4	M	75	CBS	CBD	7.4	Putamen	0	0	0	0	0	3	0	3	0	0	3	3	3	0	0	0
PiD1	M	64	CBS	Pick's	13.7	Middle/inferior frontal gyrus, anterior	0	1	0	0	3	1	0	0	0	0	3	1	3	0	0	0
PiD2	F	63	PNFA	Pick's	4.3	Middle frontal gyrus, anterior	0	0	0	0	3	1	0	0	0	0	2	2	3	0	0	0
PiD3	F	69	bvFTD	Pick's	6.3	Middle/inferior frontal gyrus, anterior	0	0	0	0	3	1	0	0	0	0	3	2	3	0	0	0
PD1	M	67	PD	PD	4.2	Anterior cingulate	0	0	0	0	0	0	0	0	0	0	0	0	0	3	3	0
PD2	F	72	PD	PD	6.1	Anterior cingulate	0	0	0	0	0	0	0	0	0	0	0	0	0	2	3	0
PD3	F	73	PD	PD	7.3	Anterior cingulate	0	0	0	0	0	0	0	0	0	0	0	0	0	3	3	0
PD4	M	69	PD	PD	5.3	Anterior cingulate	0	0	0	0	0	0	0	0	0	0	0	0	0	3	3	0
Control	F	86	Control	Control	6.4	Middle/inferior frontal gyrus, anterior	0	0	0	0	0	0	0	0	0	0	0	0	0	0	0	0

Mouse pharmacology assay for OXD-2115 and OXD-2314.

pharmidex

Summary Report

Client: Rainwater Charitable Foundation

Objective: ***Mouse PK Study for OXD2314***

Proposal Number: PPN308-002

Pharmidex Study Number: PSN22-0643

Study Performed by: DMPK

Analysis Performed by: TM

Date Study Started: 06 October 2022

Date Study Completed: 06 October 2022

Study Details

Compound:

Compound ID	MW (salt)	MW (free base)
OXD2314	-	341.39

Species (strain, sex): Mouse (NMRI, ♂) n = 3 /timepoint

Animal group numbers:

IV 1	IV 2	IV 3

Fed/Fasted: Fed

Dose Route: Intravenous

	OXD2314
Dose Level:	1 mg/kg
Dose Concentration:	0.2 mg/mL
Dose Volume:	5 mL/kg

Formulation: Solution in HP-β-CD (20% w/v) in Saline

Sampling: Serial and Terminal

Observations: No adverse events were observed

Animal Husbandry: Animals housed in pre-assigned housing cages until sampling. Appropriate samples were taken at the defined time points and stored immediately at -20°C.

Matrices Collected: Plasma Brain

Matrix: Plasma

Timepoints (hr):	0.03	0.17	0.50	1.0	2.0

Sample Preparation: Protein precipitation with acetonitrile.

Matrix: Brain

Timepoints (hr):	0.03	0.17	1.0	2.0

Sample Preparation: Protein precipitation with acetonitrile.

Sample Analysis: UHPLC - tandem mass spectrometry using electrospray ionisation.

Report by: TM

Date: Tuesday, October 25, 2022

Figure S3. Mouse pharmacology. Report summary from DMPK assay for OXD-2314.

pharmidex

PSN22-0643 In Vivo Pharmacokinetic Assessment of OXD2314 Dosed Intravenously at 1 mg/kg

[OXD2314] in Plasma					
Time (hr)	[OXD2314] ng/mL				
	IV-1	IV-2	IV-3	mean	SD
0.03	894	739	712	782	98.2
0.17	415	394	513	441	63.5
0.50	129	141	103	124	19.4
1.0	37.1	30.3	33.2	33.5	3.4
2.0	10.7	<LLOQ	<LLOQ	6.9	3.3

<LLOQ = Below Limit of Quantification, 10 ng/mL

NR = No Result

Mean and STDEV calculated using 0.5 x LLOQ where individual concentration <LLOQ

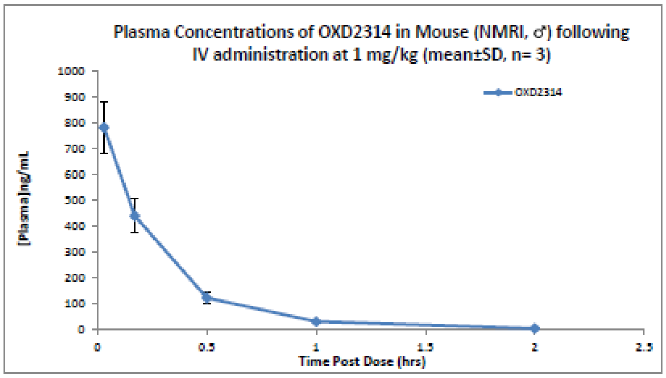

PK Parameter	Mean Value
t1/2	0.37 hr
Tmax	0.03 hr
Cmax	782 ng/mL
AUClast	263 hr*ng/mL
AUCinf	267 hr*ng/mL
Cl	62.4 mL/min/kg
Vd	1.2 L/kg
MRT	0.32 hr
C0	884 ng/mL

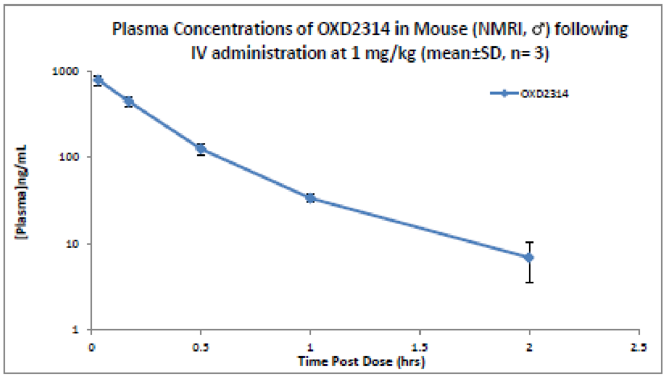

Figure S4. Mouse pharmacology. *In vivo* pharmacokinetic results in mouse plasma of OXD-2314.

pharmidex

PSN22-0643 In Vivo Pharmacokinetic Assessment of OXD2314 Dosed Intravenously at 1 mg/kg

[OXD2314] in Brain					
Time (hr)	[OXD2314] ng/g				
	IV-1	IV-2	IV-3	mean	SD
0.03	1260	1180	1120	1187	70.2
0.17	570	585	755	637	103
1.0	56.1	37.9	41.1	45.0	9.7
2.0	8.0	5.8	8.9	7.6	1.6

<LLOQ = Below Limit of Quantification, 4 ng/g

NR = No Result

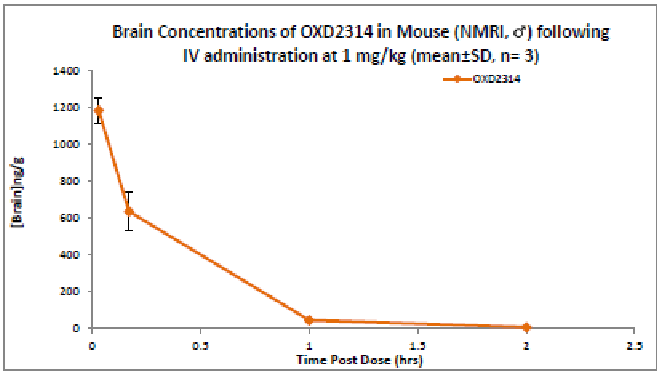

PK Parameter	Mean Value
t1/2	0.29 hr
Tmax	0.03 hr
Cmax	1187 ng/g
AUClast	455 hr*ng/g
AUCinf	458 hr*ng/g

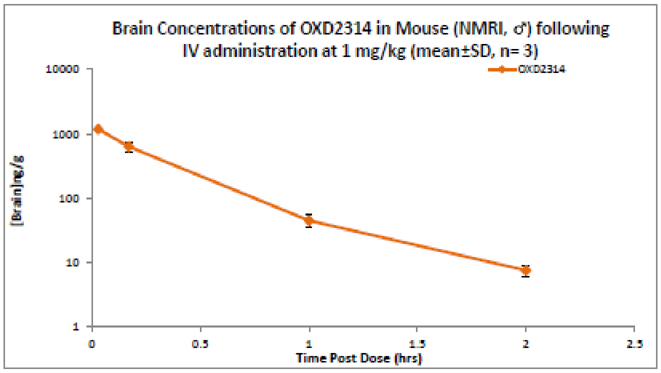

Figure S5. Mouse pharmacology. *In vivo* pharmacokinetic results in mouse brain of OXD-2314.

pharmidex

Summary Report

Client: Rainwater Charitable Foundation
Objective: ***Mouse PK Study for OXD2115***
Proposal Number: PPN308-002
Study Performed by: DMPK
Date Study Started: 06 October 2022

Pharmidex Study Number: PSN22-0642
Analysis Performed by: TM
Date Study Completed: 06 October 2022

Study Details

Compound:

Compound ID	MW (salt)	MW (free base)
OXD2115	-	341.39

Species (strain, sex): Mouse (NMRI, ♂) n = 3 /timepoint

Animal group numbers:

IV 1	IV 2	IV 3

Fed/Fasted: Fed

Dose Route: Intravenous

	OXD2115
Dose Level:	1 mg/kg
Dose Concentration:	0.2 mg/mL
Dose Volume:	5 mL/kg

Formulation: Solution in HP-β-CD (20% w/v) in Saline

Sampling: Serial and Terminal

Observations: No adverse events were observed

Animal Husbandry: Animals housed in pre-assigned housing cages until sampling. Appropriate samples were taken at the defined time points and stored immediately at -20°C.

Matrices Collected: Plasma Brain

Matrix: Plasma

Timepoints (hr):					
	0.03	0.17	0.50	1.0	2.0

Sample Preparation: Protein precipitation with acetonitrile.

Matrix: Brain

Timepoints (hr):				
	0.03	0.17	1.0	2.0

Sample Preparation: Protein precipitation with acetonitrile.

Sample Analysis: UHPLC - tandem mass spectrometry using electrospray ionisation.

Report by: TM Date: Wednesday, October 26, 2022

Figure S6. Mouse pharmacology. Report summary from DMPK assay for OXD-2115.

pharmidex

PSN22-0642 In Vivo Pharmacokinetic Assessment of OXD2115 Dosed Intravenously at 1 mg/kg

[OXD2115] in Plasma					
Time (hr)	[OXD2115] ng/mL				
	IV-1	IV-2	IV-3	mean	SD
0.03	823	1900	1040	1254	570
0.17	292	226	256	258	33.0
0.50	<LLOQ	<LLOQ	<LLOQ		
1.0	<LLOQ	<LLOQ	<LLOQ		
2.0	<LLOQ	<LLOQ	<LLOQ		

<LLOQ = Below Limit of Quantification, 20 ng/mL

NR = No Result

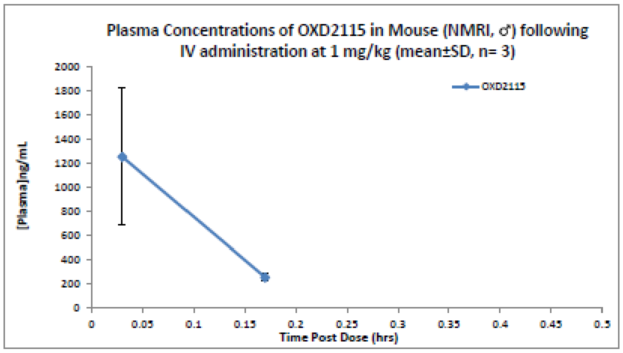

Mean and STDEV calculated using 0.5 x LLOQ where individual concentration <LLOQ

PK Parameter	Mean Value
t1/2	nr hr
Tmax	0.03 hr
Cmax	1254 ng/mL
AUClast	151 hr*ng/mL
AUCinf	nr hr*ng/mL
Cl	nr mL/min/kg
Vd	nr L/kg
MRT	nr hr
C0	1760 ng/mL

nr = No Result; cannot be determined due to lack of time points

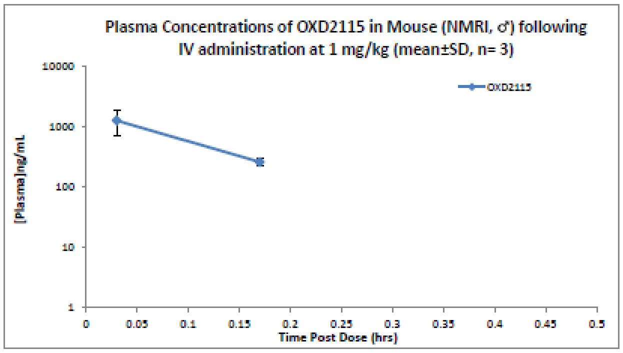

Figure S7. Mouse pharmacology. *In vivo* pharmacokinetic results in mouse plasma of OXD-2115.

pharmidex

PSN22-0642 In Vivo Pharmacokinetic Assessment of OXD2115 Dosed Intravenously at 1 mg/kg

[OXD2115] in Brain					
Time (hr)	[OXD2115] ng/g				
	IV-1	IV-2	IV-3	mean	SD
0.03	131	95.2	185	137	45.2
0.17	43.1	52.2	49.1	48.1	4.6
1.0	<LLOQ	<LLOQ	<LLOQ		
2.0	<LLOQ	<LLOQ	<LLOQ		

<LLOQ = Below Limit of Quantification, 2 ng/g

NR = No Result

Mean and STDEV calculated using 0.5 x LLOQ where individual concentration <LLOQ

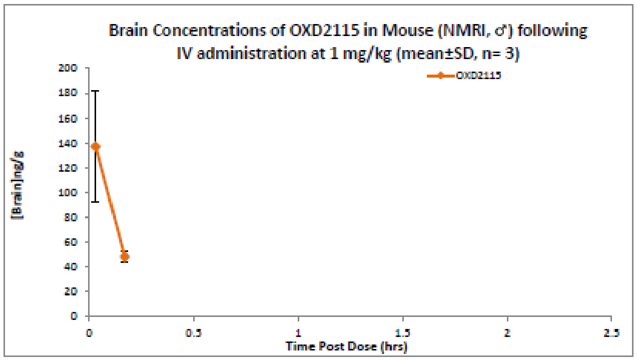

PK Parameter	Mean Value
t1/2	nr hr
Tmax	0.03 hr
Cmax	137 ng/g
AUClast	15.0 hr*ng/g
AUCinf	nr hr*ng/g

nr = No Result; cannot be determined due to lack of time points

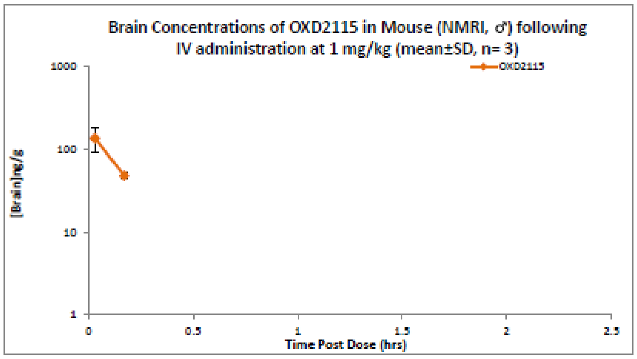

Figure S8. Mouse pharmacology. *In vivo* pharmacokinetic results in mouse brain of OXD-2115.

Table S2. Mouse pharmacology. Ratio of brain and plasma concentrations of OXD-2314 (brain:plasma) following *i.v.* administration. Area under the curve (AUC).

	TOTAL		FREE	
	AUC	brain:plasma	AUC	brain:plasma
Plasma	267		0.160	
Brain	458	1.7	0.092	0.6

Table S3. Mouse pharmacology. Ratio of brain and plasma concentrations of OXD-2115 following *i.v.* administration.

	TOTAL		FREE	
	AUC	brain:plasma	AUC	brain:plasma
Plasma	151		14	
Brain	15	0.1	0.030	0.002

***In vitro* pharmacology screening performed by Eurofins**.

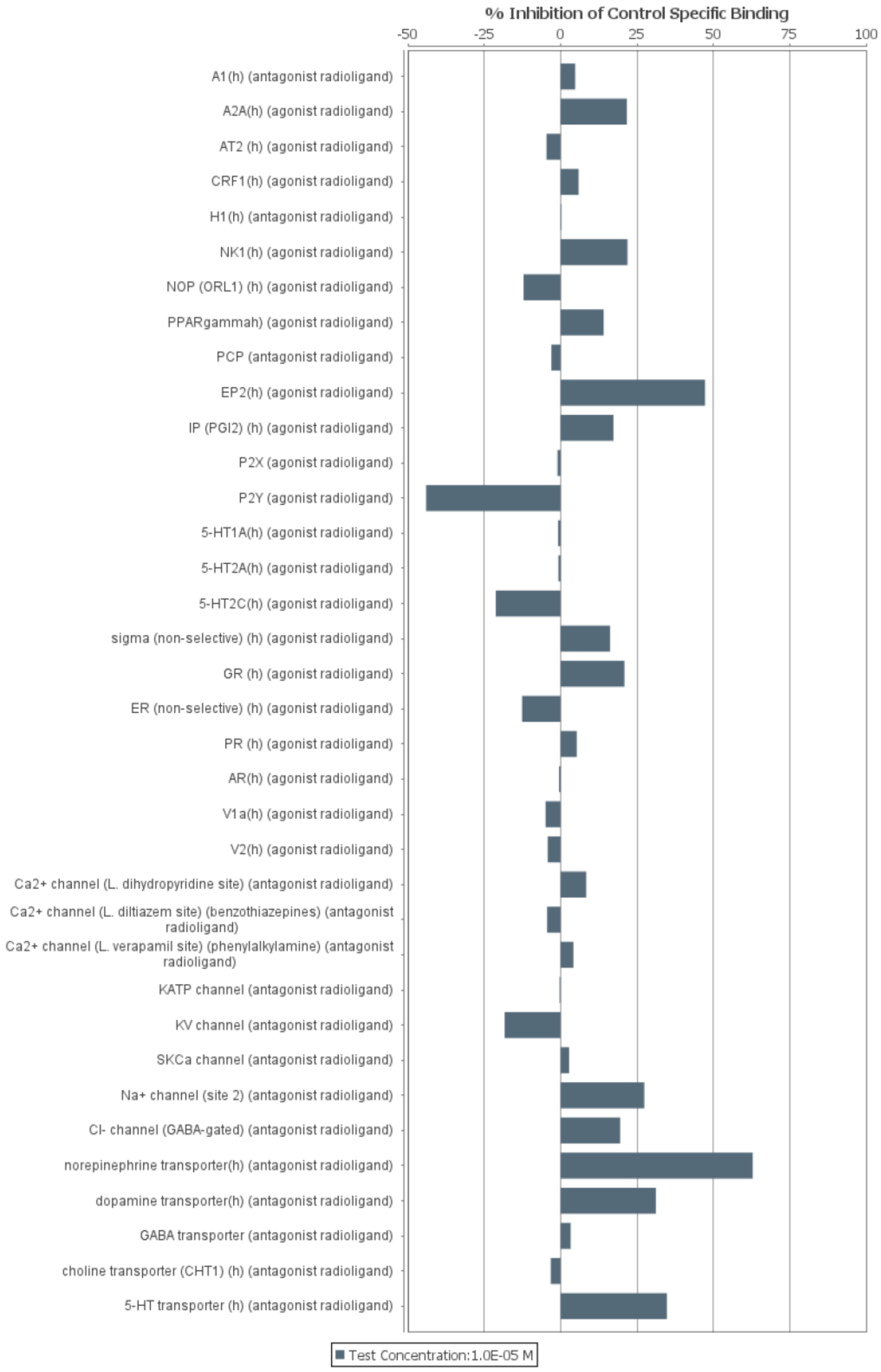

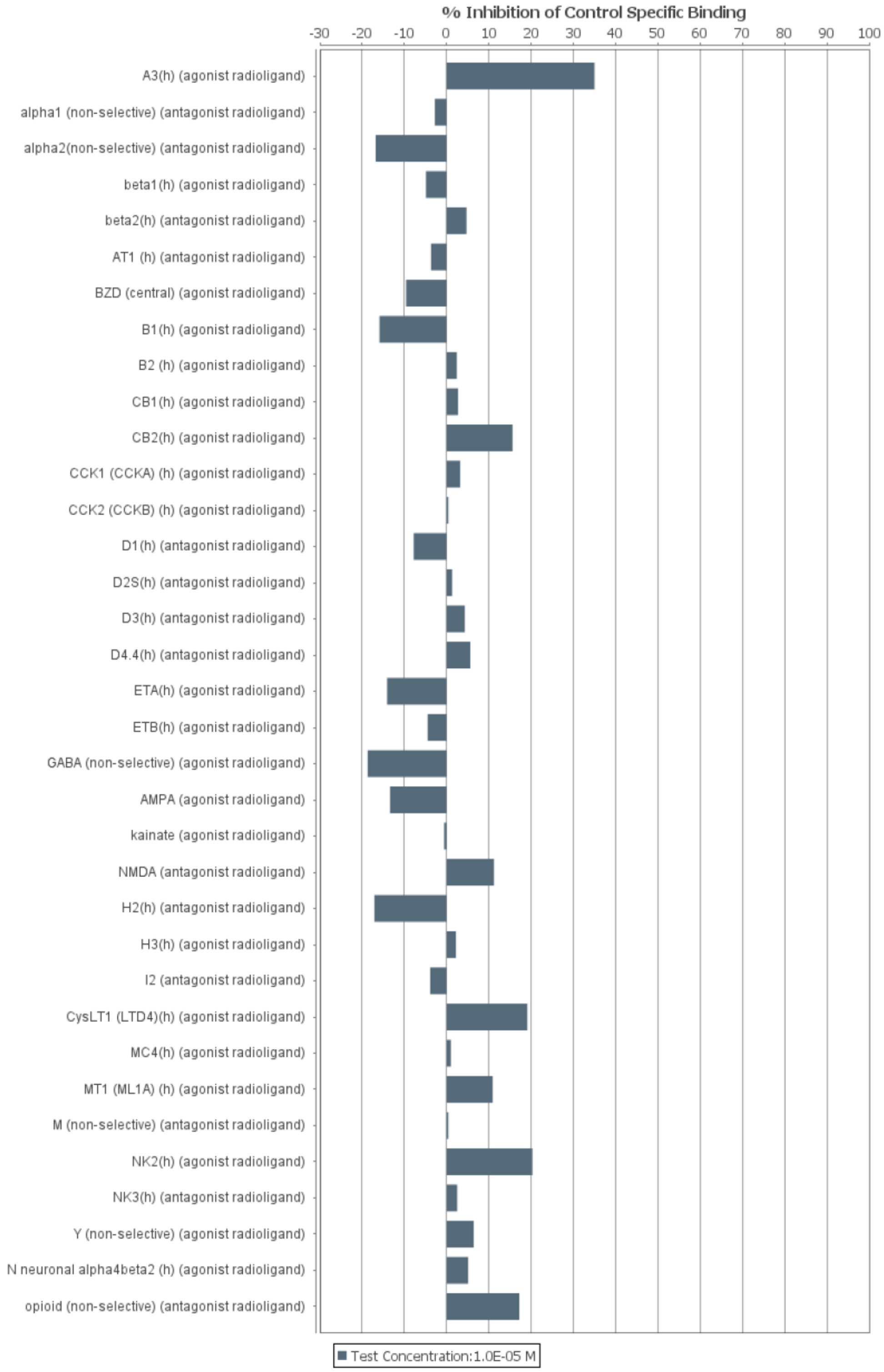

Figure S9. *In vitro* pharmacology of OXD-2314. %Inhibition of radioligand binding to CNS receptors by OXD-2314 at 10μM.

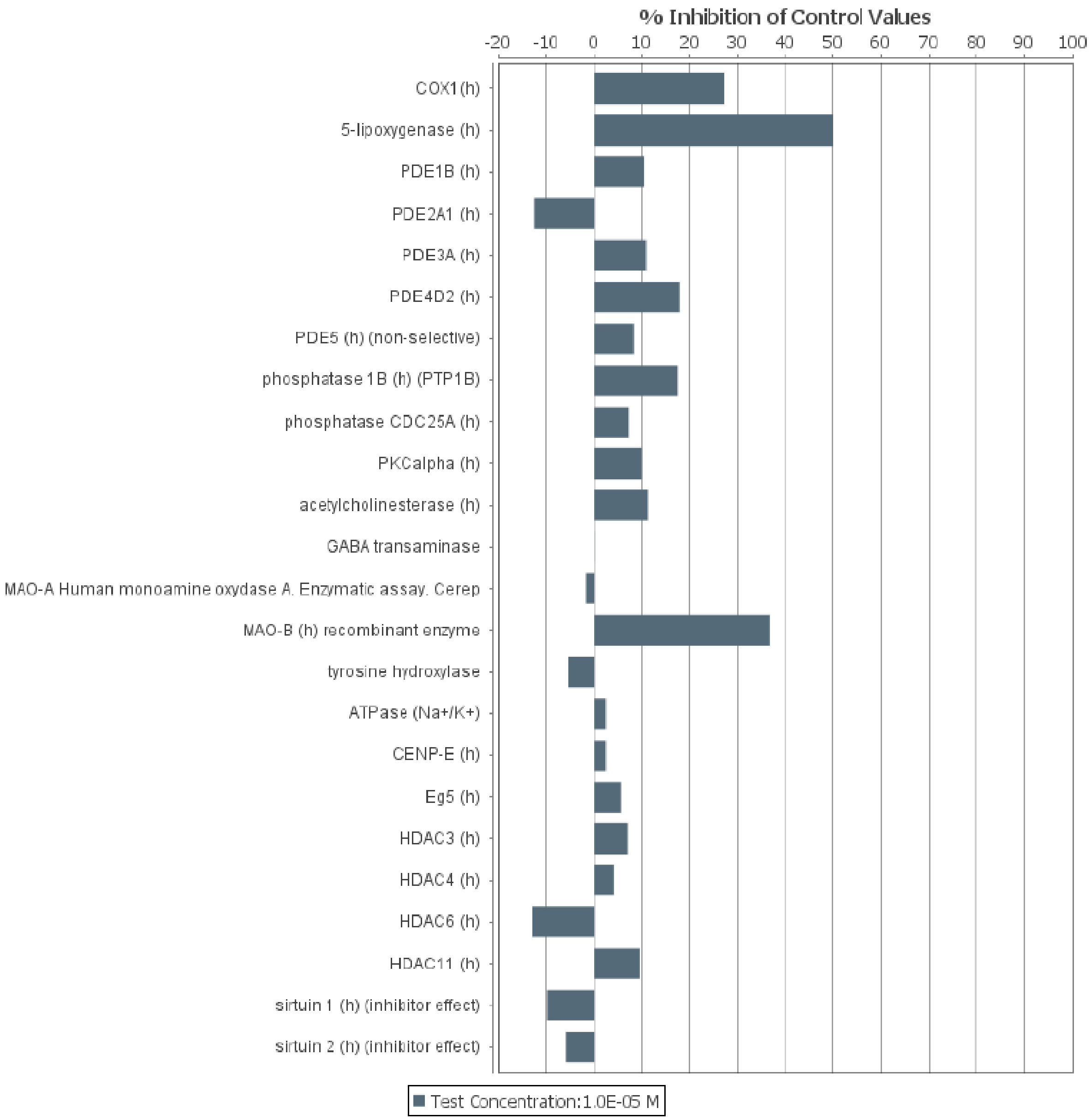

Figure S10. *In vitro* pharmacology of OXD-2314. CNS enzyme inhibition by OXD-2314 at 10µM.

Table S4. Blocking study using [^{3}H]OXD-2314 in AD, PSP and CBD tissue against MAO-A and MAO-B substrates deprenyl and Ro41-1049.

**Radioligand**	**Cold Compound**	**Tissue**	**K_i (nM)**
[^{3}H]OXD-2314	Deprenyl	AD (*n* = 1)	2900
[^{3}H]OXD-2314	Deprenyl	PSP (*n* = 1)	3000
[^{3}H]OXD-2314	Deprenyl	CBD (*n* = 1)	5000
[^{3}H]OXD-2314	Ro41-1049	AD (*n* = 1)	>10,000
[^{3}H]OXD-2314	Ro41-1049	PSP (*n* = 1)	>10,000
[^{3}H]OXD-2314	Ro41-1049	CBD (*n* = 1)	>10,000

Radiometabolite analysis in rats.

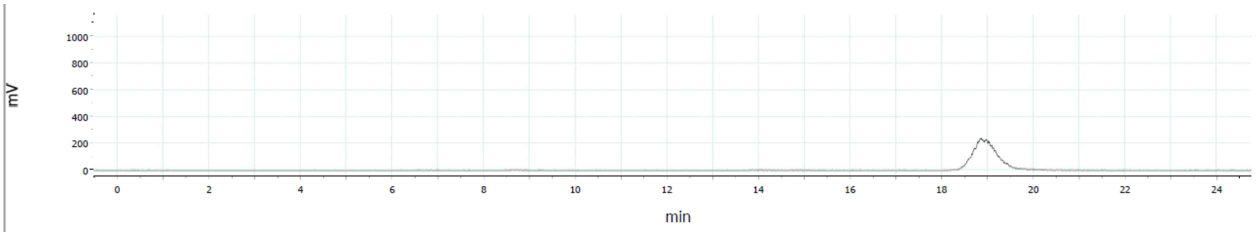

Figure S11. Basline HPLC chromatogram of standard [^{18}F]OXD-2314 for metabolite studies in rat. UV signal top.

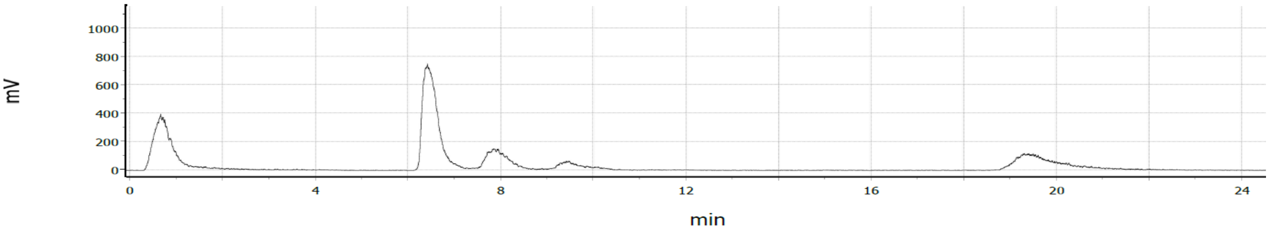

Figure S12. Radiometabolite study. HPLC chromatogram of blood plasma from female rat following *i.v.* administration of [^{18}F]OXD-2314. t=30 min post-injection.

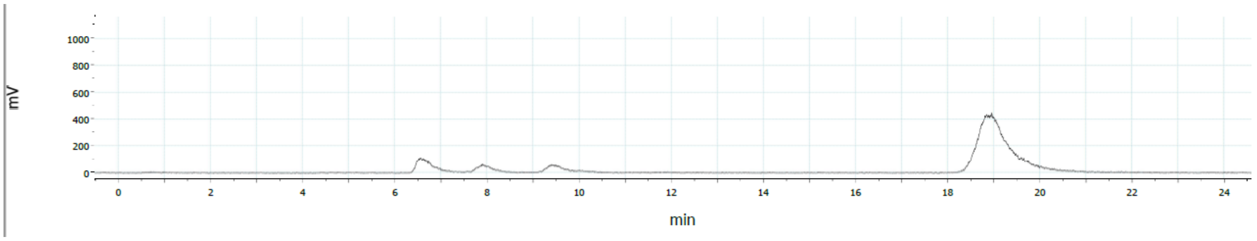

Figure S13. Radiometabolite study. HPLC chromatogram of brain homogenate sample from female rat following *i.v.* administration of [^{18}F]OXD-2314. t=30 min post injection

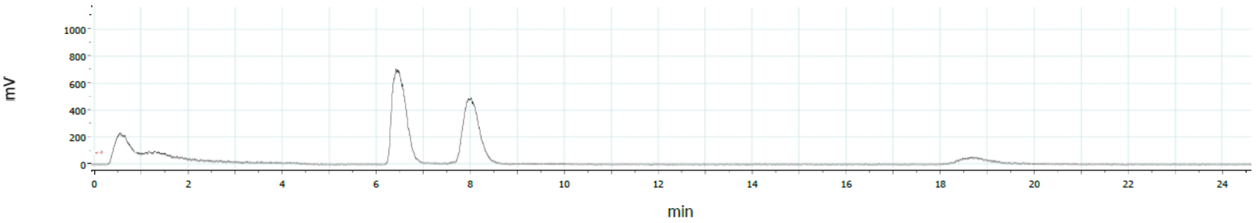

Figure S14. Radiometabolite study. HPLC chromatogram of blood plasma from male rat following *i.v.* administration of [^{18}F]OXD-2314. t=30 min post injection

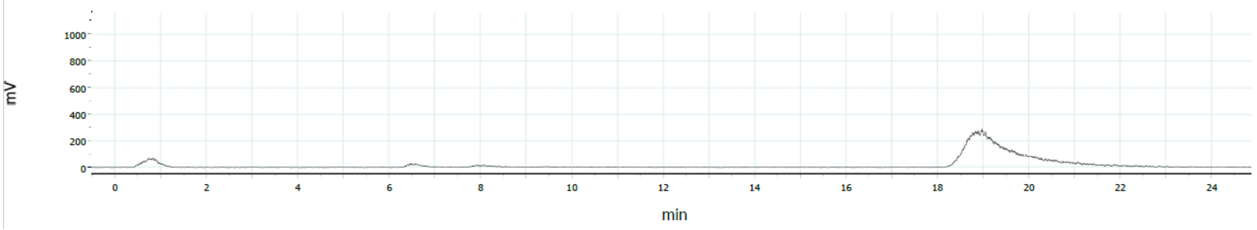

Figure S15. Radiometabolite study. HPLC chromatogram of brain homogenate sample from male rat following *iv*. administration of [^{18}F]OXD-2314. t=30 min post injection.

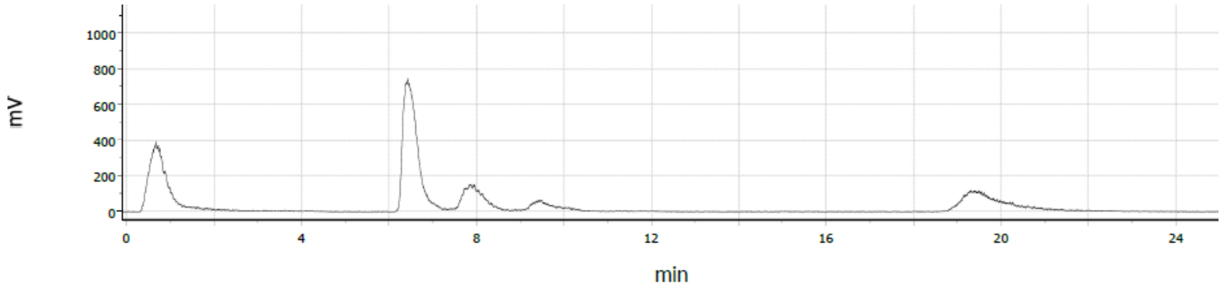

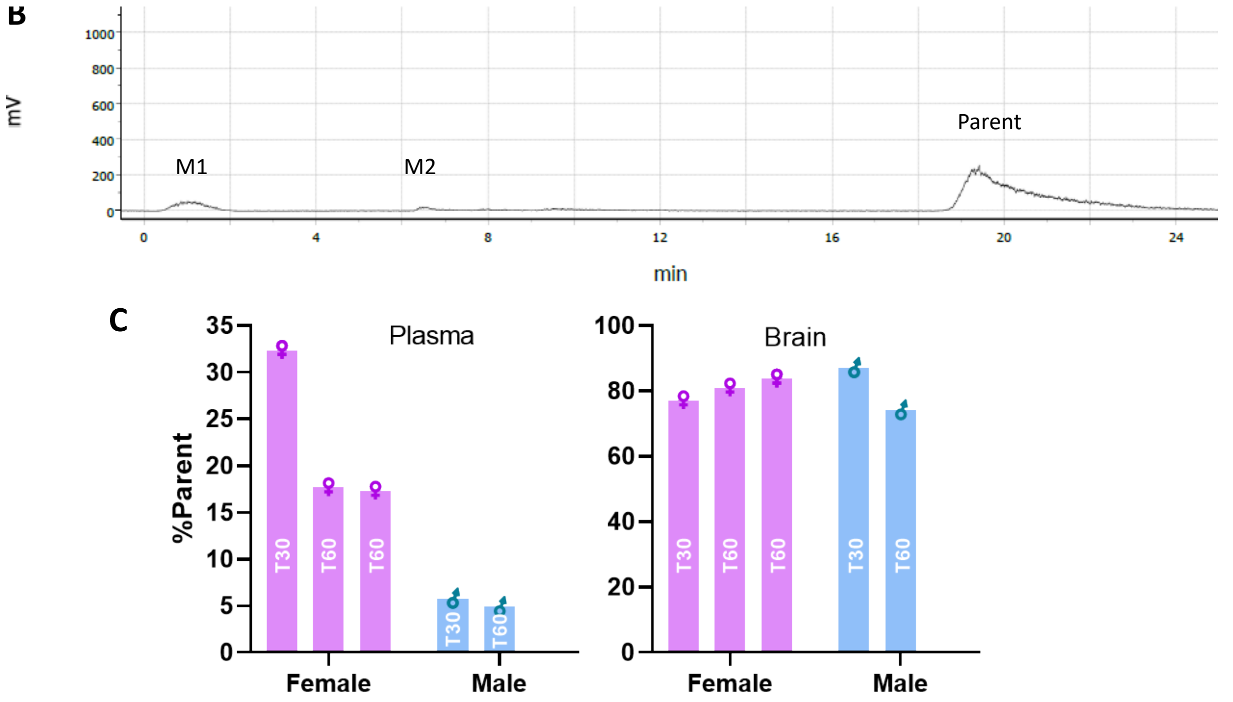

Figure S16. Radiometabolite analysis in plasma and brain. Representative column-switch HPLC chromatograms are shown for female rat plasma (**A**) and brain (**B**), with the parent [^{18}F]OXD-2314 and four radiometabolites (M1-M4) indicated. (**C**) shows the parent fraction (%) in plasma and brain of male (n=2) and female (n=3) rats at 30 min (T30) and 60 min (T60) post-radiotracer injection. Note that the rats were not perfused with saline prior to death, thereby likely resulting in the trapping of radiometabolites contained in blood in the brains of the rats.

Dosimetry analysis in rats.

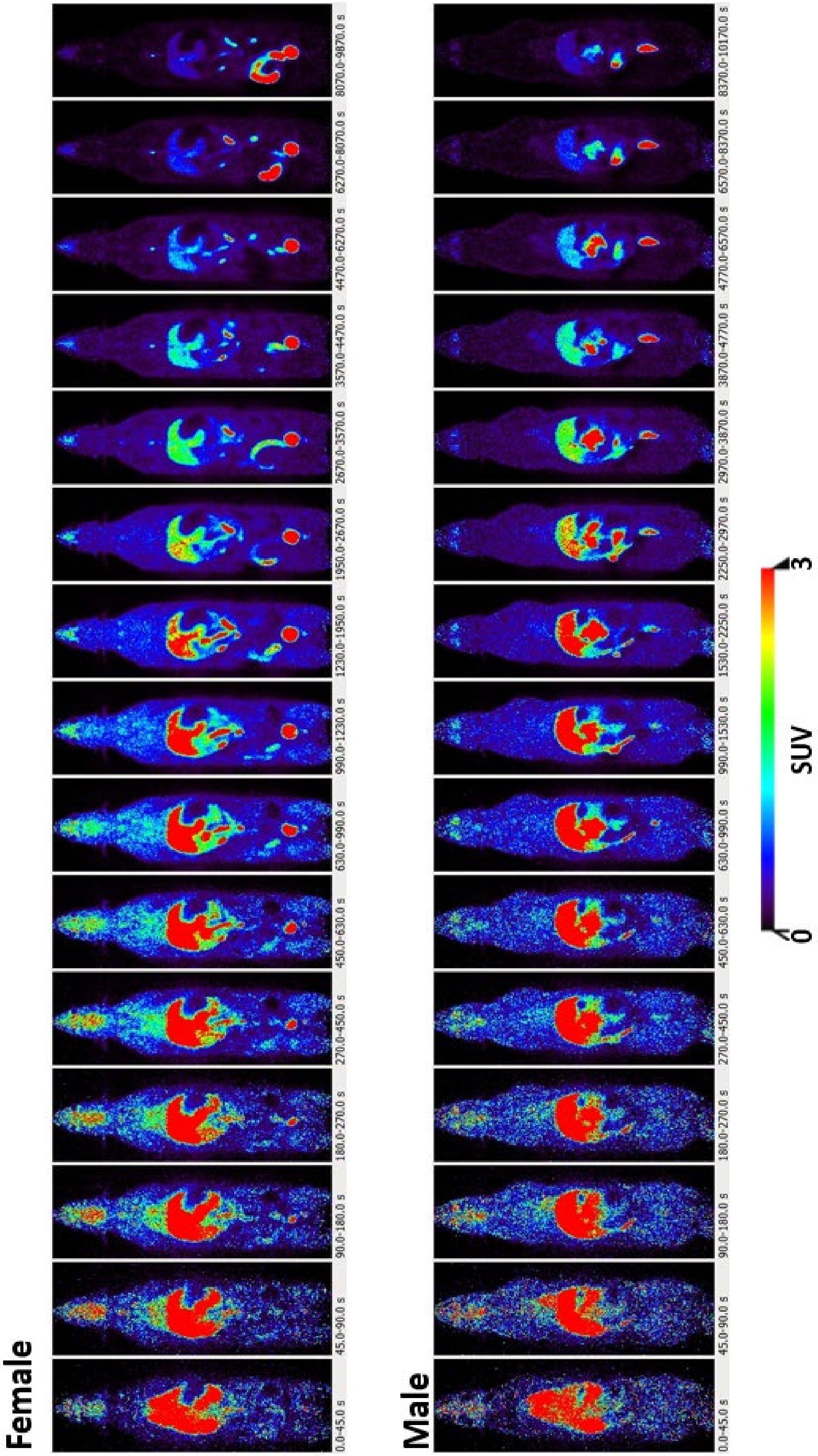

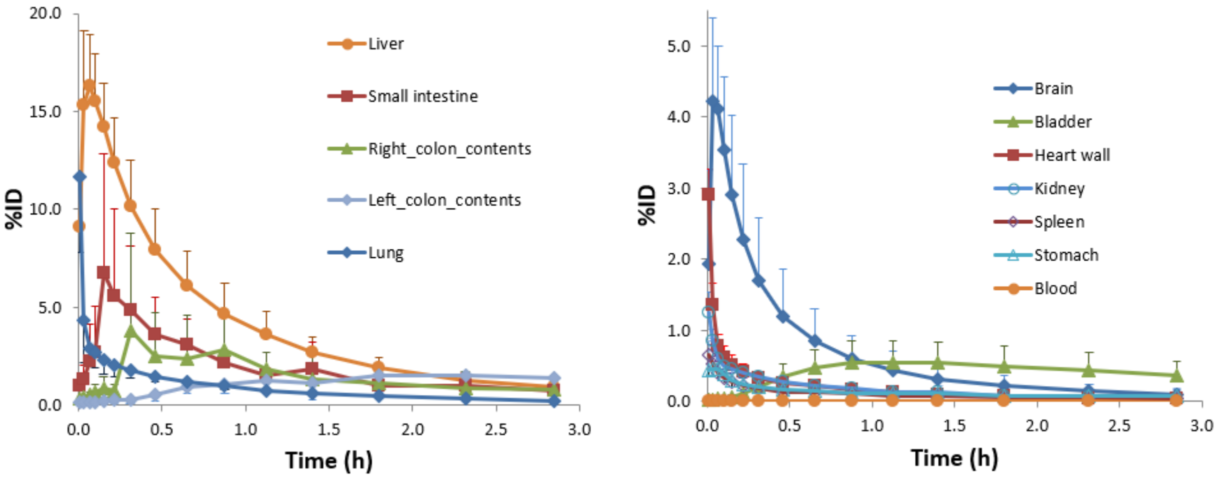

Figure S17. Dosimetry in rats. Representative full body images of 15-time frames from female and male rat PET scans of [^{18}F]OXD-2314 and organ TACs from rat full-body PET scans of [^{18}F]OXD-2314.

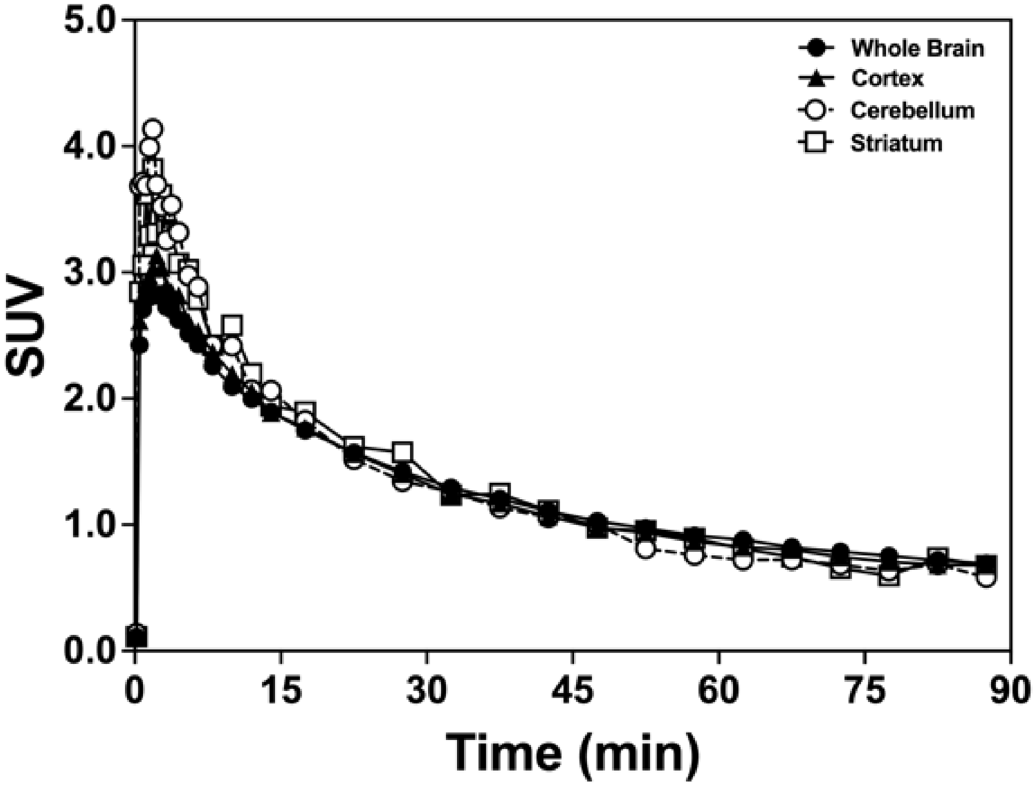

Figure S18. Baboon PET scan. Time activity curves in a male baboon following [^{18}F]OXD-2314 injection showing no preferential radiotracer retention in striatum.

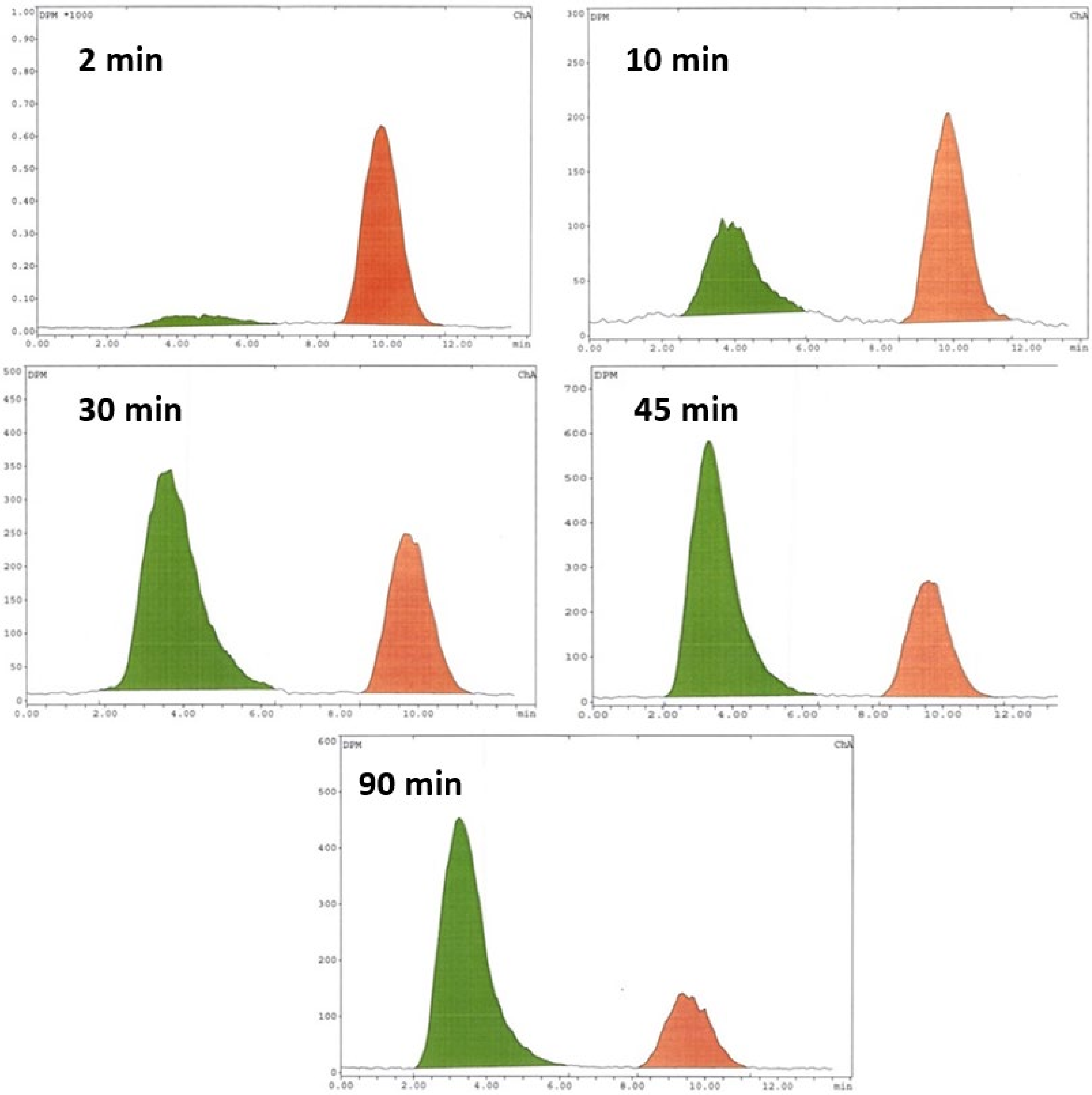

Figure S19. Radiometabolite study. HPLC chromatogram of venous plasma samples from male NHP following *i.v.* administration of [^{18}F]OXD-2314 taken at 2, 10, 30, 45 and 90 min post injection. Unchanged parent [^{18}F]OXD-2314 shown in orange (R_t 8-12 min), radiometabolite fraction shown in green(R_t 2-6 min).

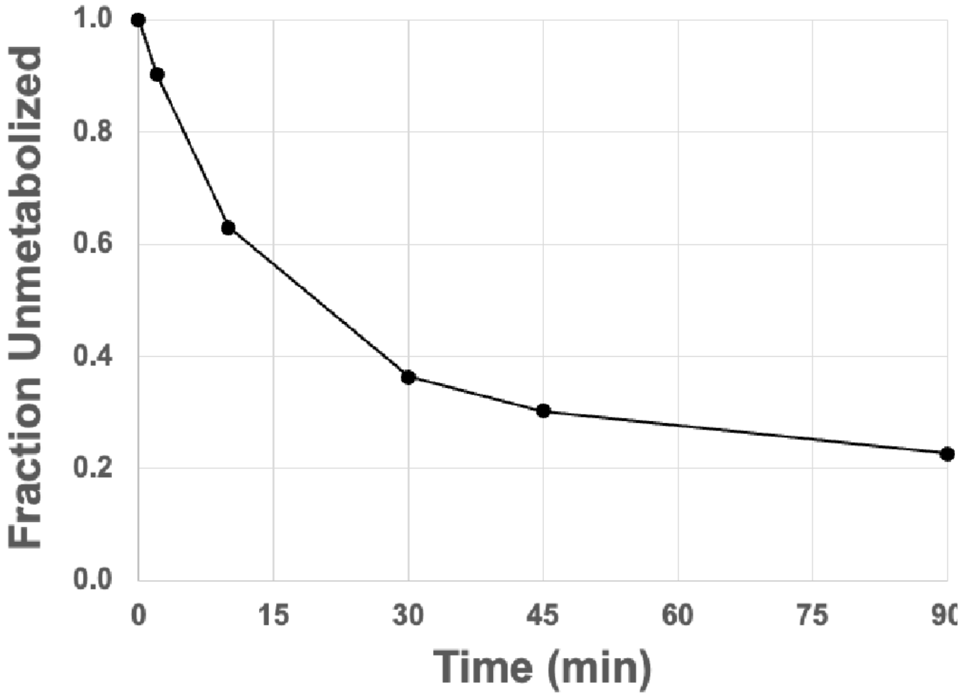

Figure S20. Radiometabolite study. Fraction of unmetabolized [^{18}F]OXD-2314 in venous male NHP plasma over 90 min post *i.v.* injection.

Autoradiography using [^{3}H]OXD-2314

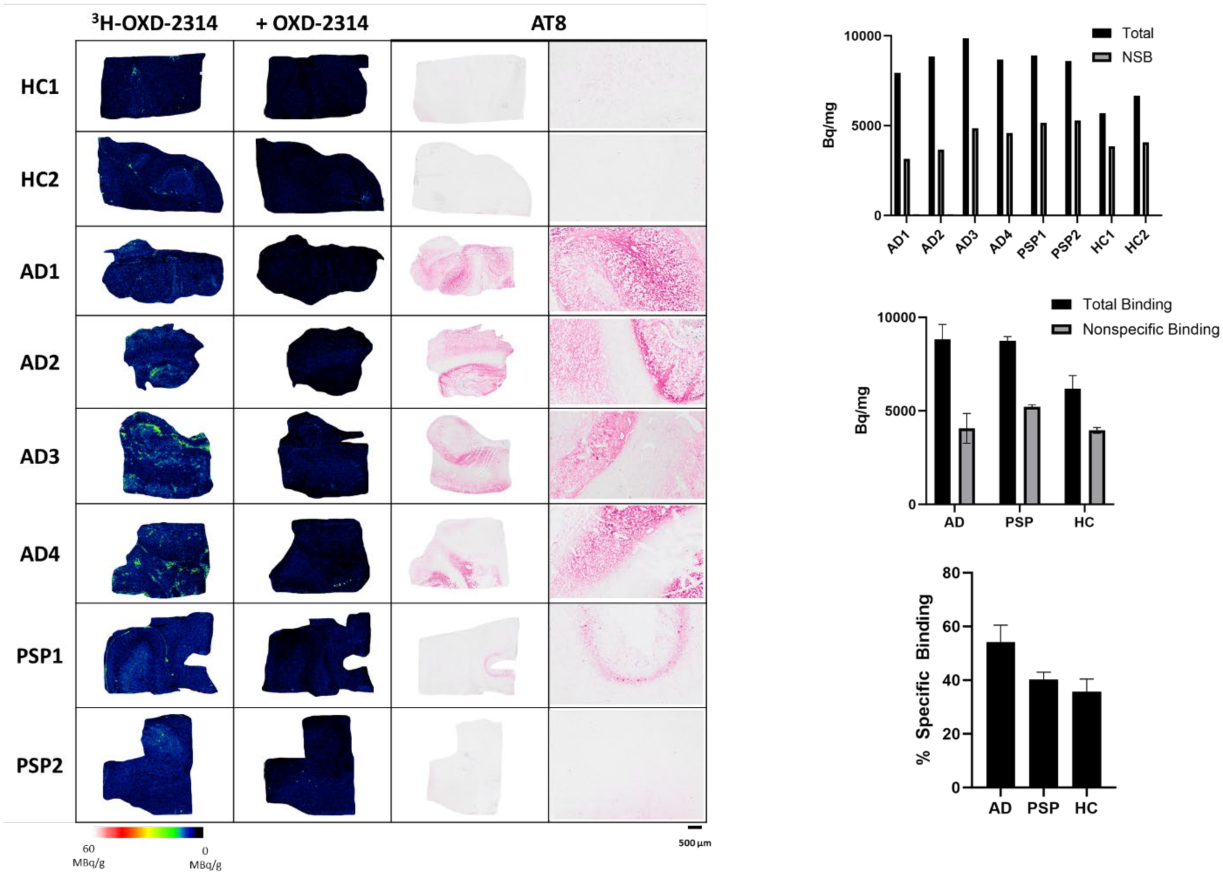

Figure S21. Autoradiography. [^{3}H]OXD-2314 binding in HC, AD and PSP hippocampal tissue. Total [^{3}H]OXD-2314 (3 nM) binding is shown along with displacement by unlabeled OXD-2314 (10 μM) compared to AT8 immunostaining for tau. Low specific binding was observed in aged healthy control tissues and PSP tissues with low tau distribution, whereas elevated radiotracer signal and good specific binding was observed in hippocampal AD, as confirmed by IHC staining for phospho-tau.

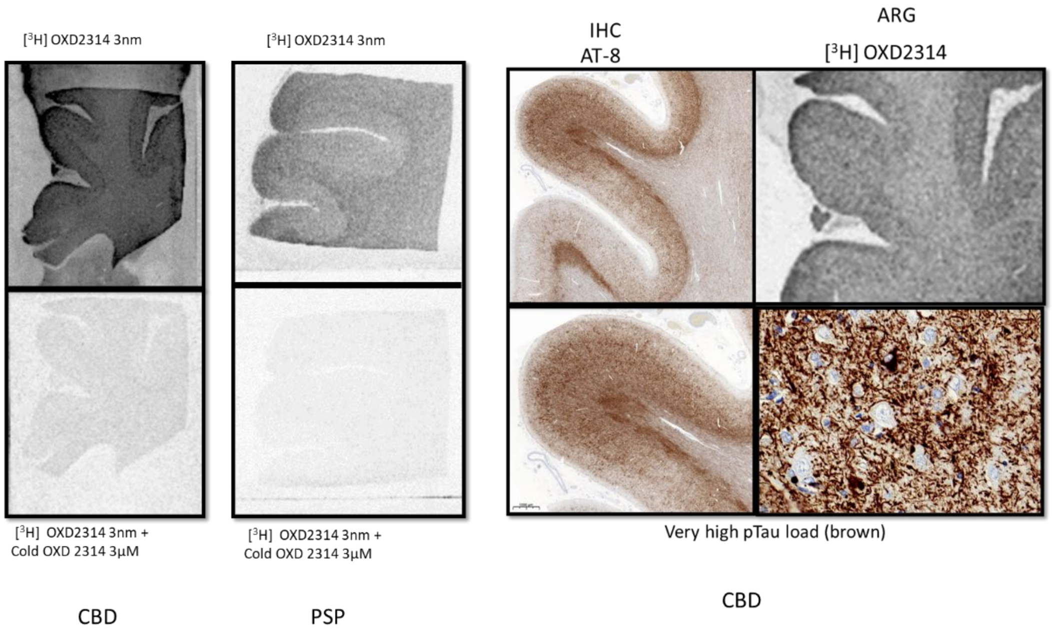

Figure S22. Autoradiography. [^{3}H]OXD-2314 binding in CBD and PSP cortical tissue. Total [^{3}H]OXD-2314 (3 nM) binding is shown along with displacement by unlabeled OXD-2314 (3 μM) and AT8 immunostaining for tau in CBD tissue.

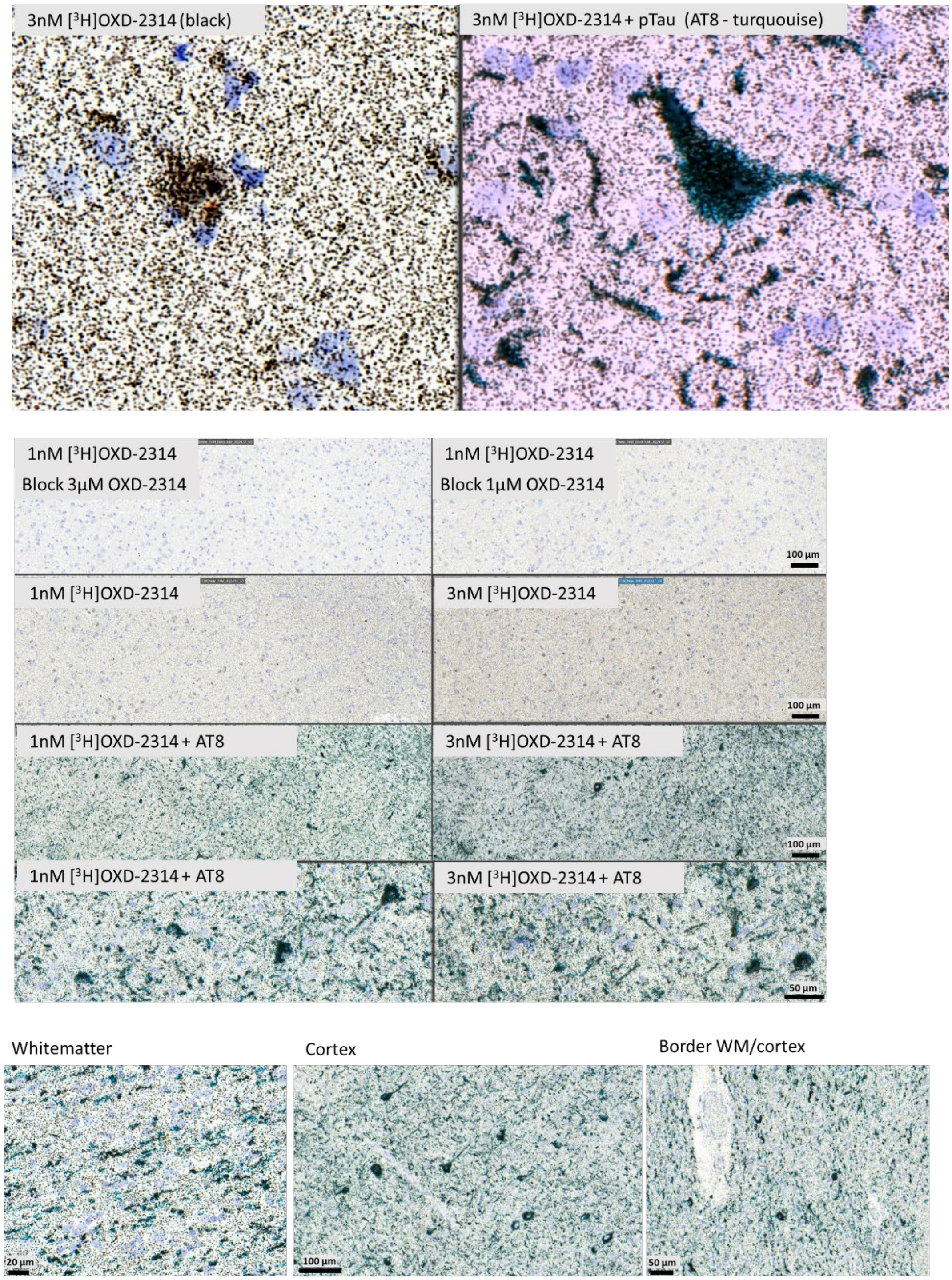

Figure S23. Autoradiography. [^{3}H]OXD-2314 high resolution autoradiography in CBD cortical tissue. [^{3}H]OXD-2314 signal (1 or 3 nM) (black) is overlaid with AT8 staining for phospho-tau (turquoise).

Table S5. Human tissue data. Tissue Demographics used in autoradiography with [^{3}H]OXD-2314.

Primary Pathological Diagnosis	Case ID	Region	Sex	Age
HC; A0B1C0; Low NFT	HC1	BA28/HP	F	72
Healthy	HC2	BA28/HP	F	51
AD; A3B2C3	AD1	BA28/HP	M	90
AD; A3B3C3	AD2	BA28/HP	M	88
AD; A3B3C3	AD3	BA28/HP	F	93
AD; Braak IV/V	AD4	BA28/HP	F	96
PSP	PSP1	BA28/HP	M	65
PSP	PSP2	BA28/HP	F	72

*BA: Brodmann's area; M: male; F: female

Representative homologous binding assay results of [^{3}H]OXD-2314

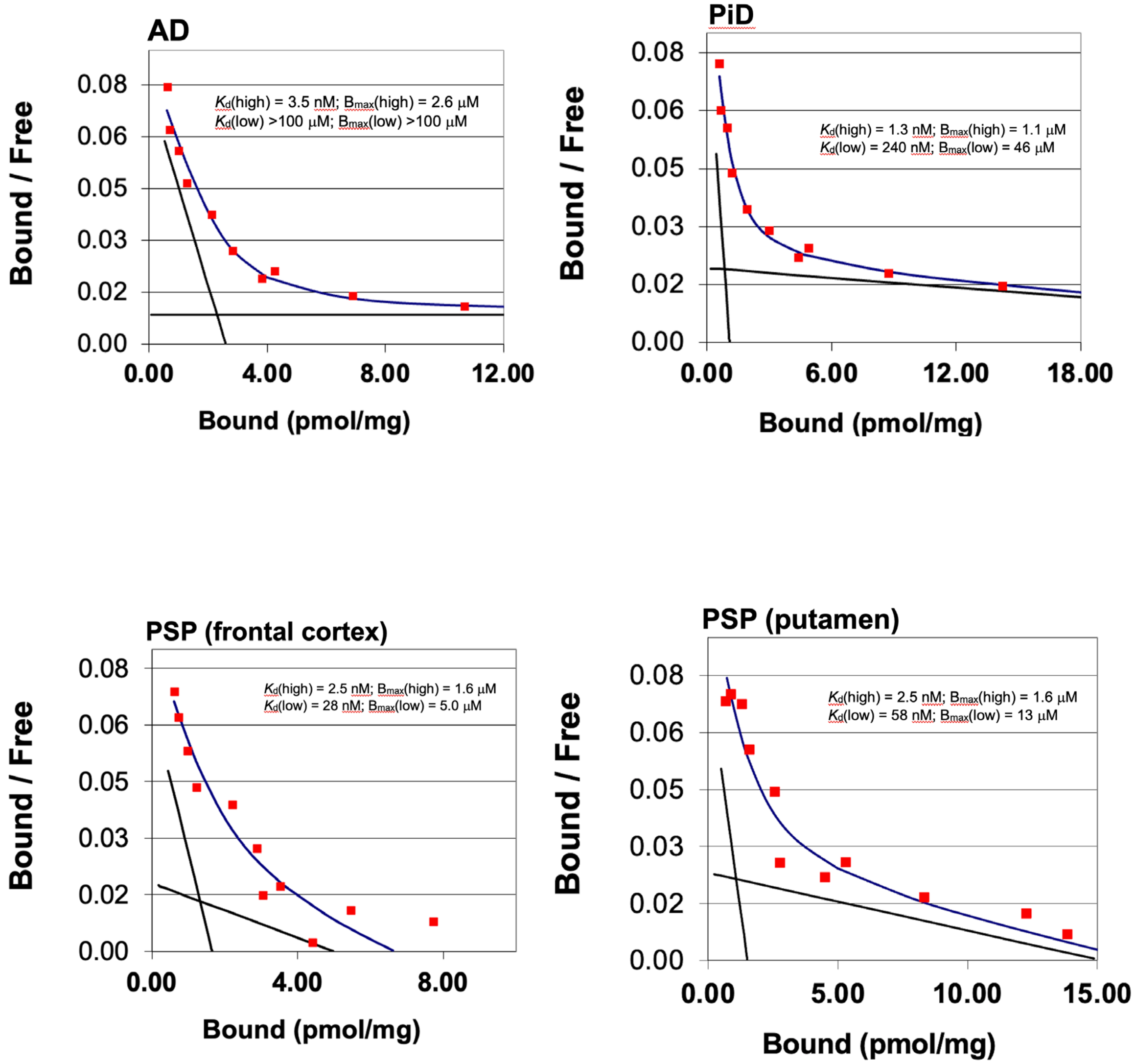

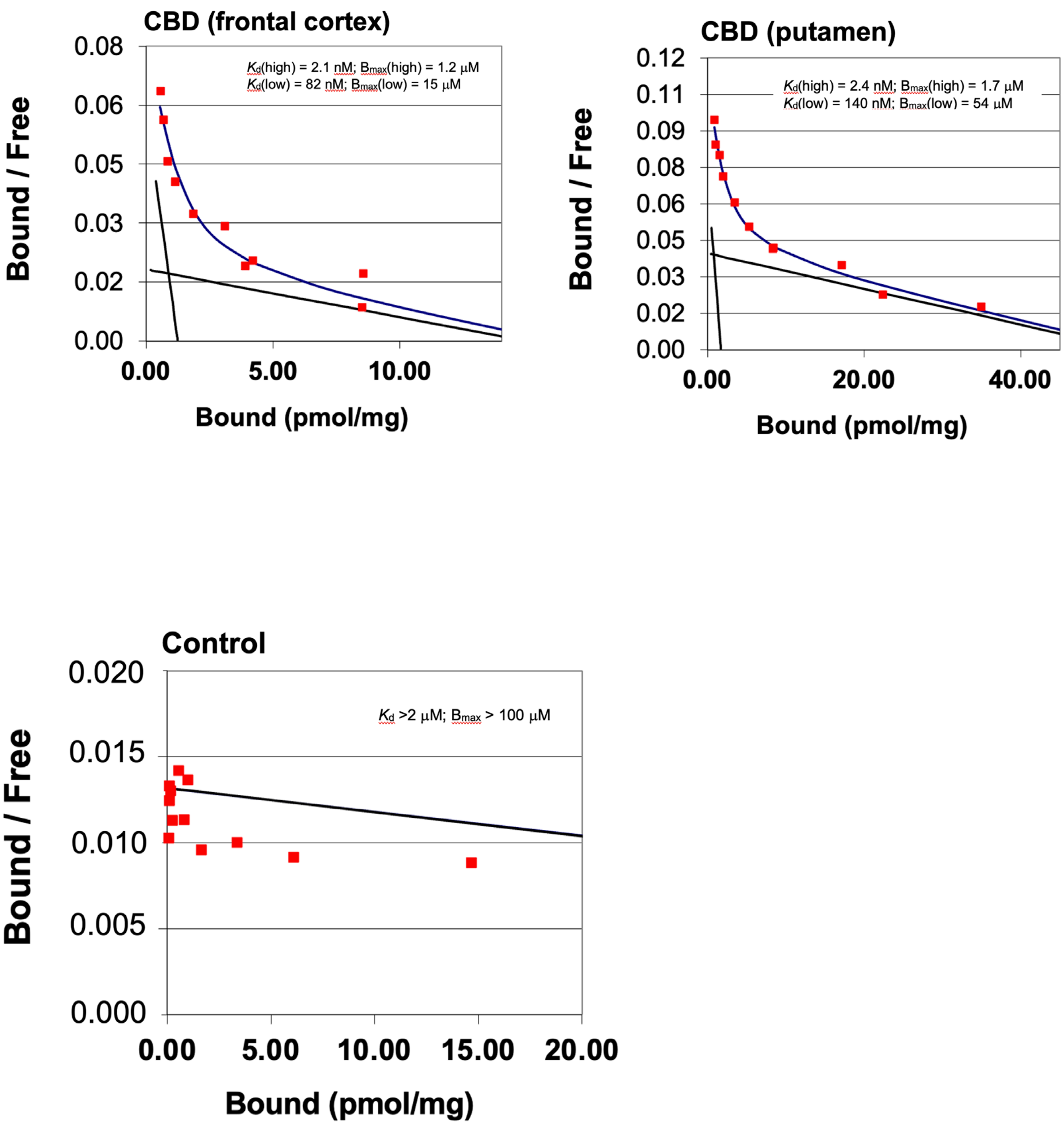

Figure S24. *In vitro* characterization. Representative homologous binding assay results of [^{3}H]OXD-2314 in AD, PiD, PSP, CBD, and control brain tissues. Two-site binding (high and low affinity) was identified in all brain tissues except control.

^{13}C- and ^{1}H-NMR spectra for compounds 1-8 including OXD-2314.

2-Fluoro-6-[(3*S*)-3-methoxypiperidin-1-yl]pyridine (1)

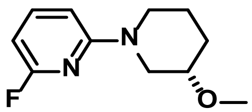

^{13}C NMR (126 MHz, dmso) δ 163.41, 161.56, 158.28, 158.15, 142.90, 142.83, 103.56, 103.52, 94.90, 94.61, 74.64, 55.93, 48.37, 45.00, 29.95, 22.09.

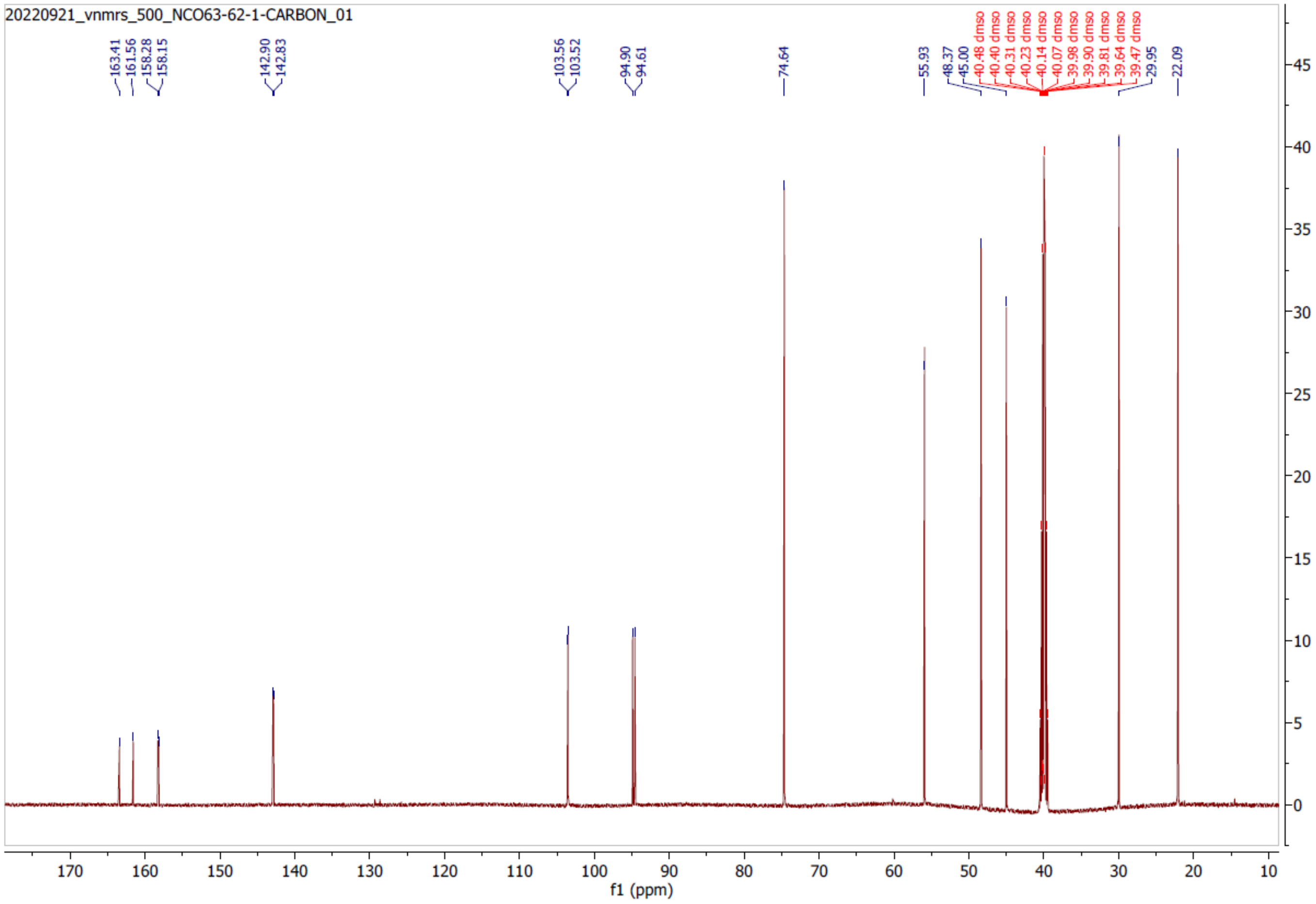

Figure S25. ^{13}C-NMR of 2-Fluoro-6-[(3S)-3-methoxypiperidin-1-yl]pyridine (1)

^{1}H NMR (500 MHz, dmso) δ 7.59 (ddd, *J* = 9.2, 8.3, 7.7 Hz, 1H), 6.69 – 6.60 (m, 1H), 6.16 (ddd, *J* = 7.7, 2.9, 0.4 Hz, 1H), 3.86 (dq, *J* = 11.0, 1.3 Hz, 1H), 3.64 (dddt, *J* = 13.2, 5.6, 3.9, 0.9 Hz, 1H), 3.25 (s, 3H), 3.24 – 3.15 (m, 3H), 1.95 – 1.84 (m, 1H), 1.68 (dddd, *J* = 13.3, 7.2, 6.0, 3.5 Hz, 1H), 1.52 – 1.43 (m, 1H), 1.39 (dtt, *J* = 13.2, 9.2, 3.7 Hz, 1H).

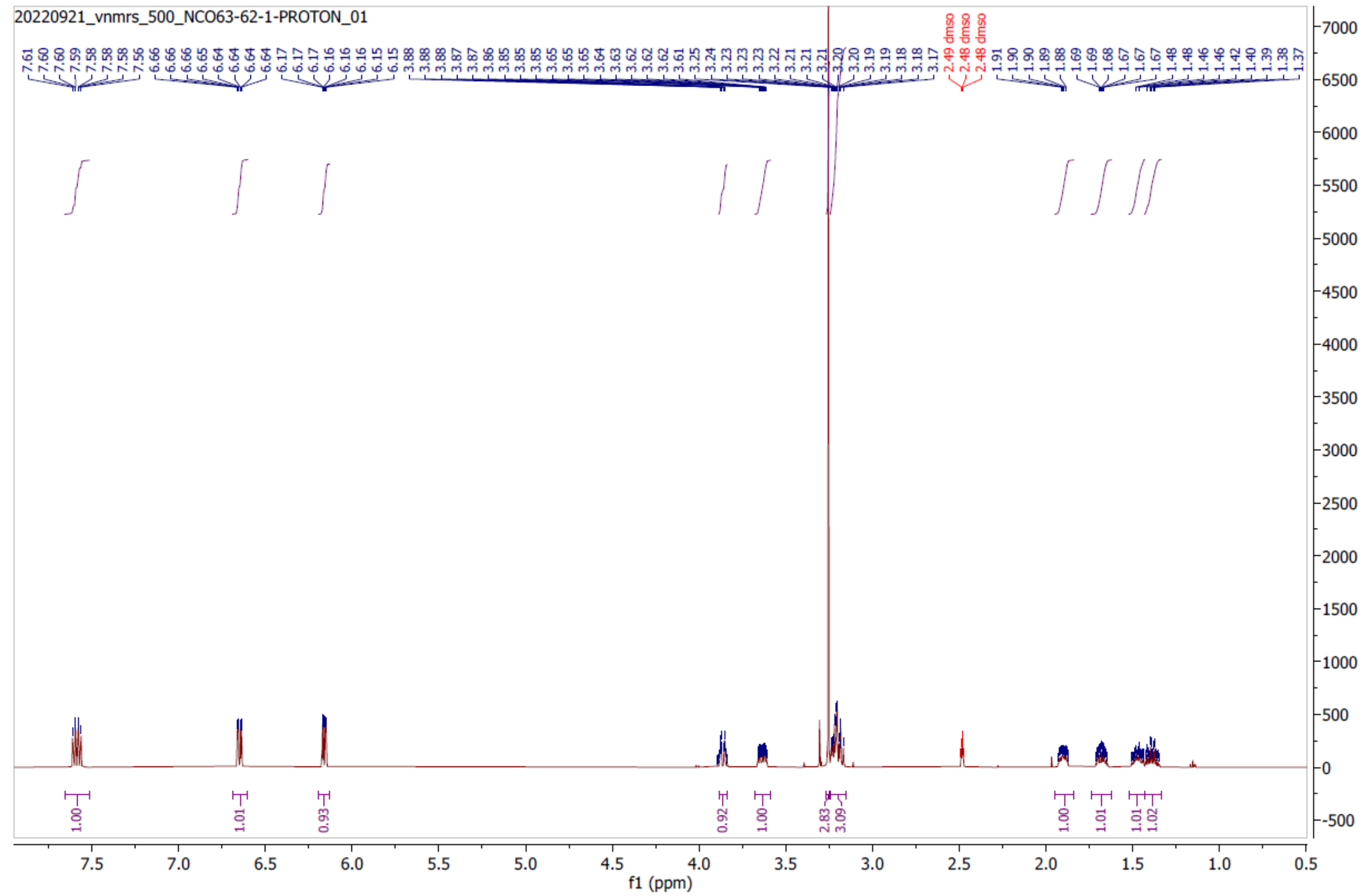

Figure S26. ^{1}H-NMR of 2-Fluoro-6-[(3S)-3-methoxypiperidin-1-yl]pyridine (1)

3-Bromo-2-fluoro-6-[(3*S*)-3-methoxypiperidin-1-yl]pyridine (2)

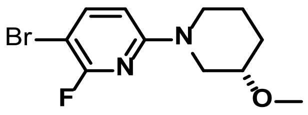

^{13}C NMR (126 MHz, dmso) δ 158.61, 157.07, 156.95, 156.78, 144.96, 144.94, 106.04, 106.01, 85.85, 85.54, 74.51, 55.98, 48.40, 45.16, 29.70, 21.85.

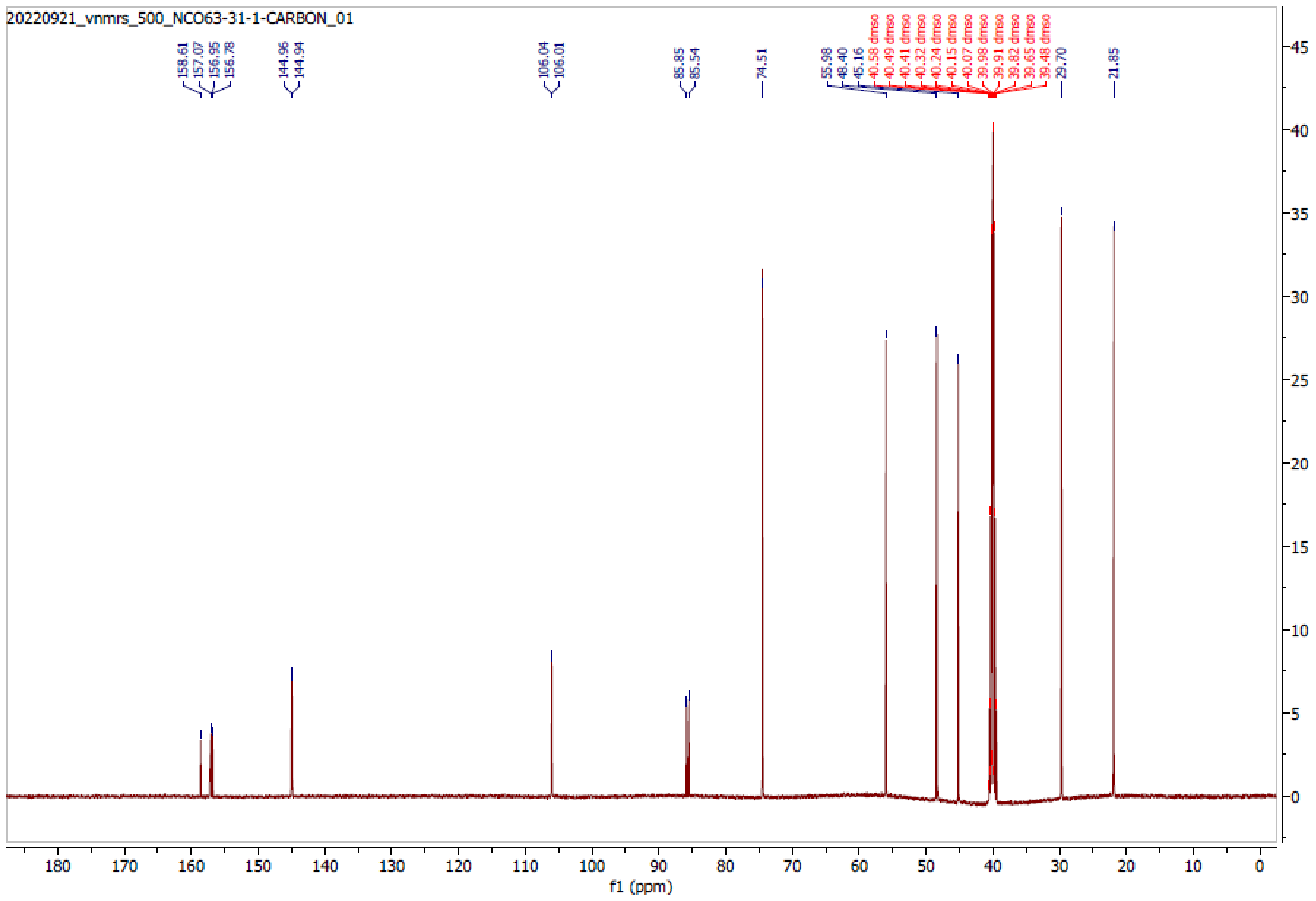

Figure S27. ^{13}C-NMR of 3-Bromo-2-fluoro-6-[(3*S*)-3-methoxypiperidin-1-yl]pyridine (2)

^{1}H NMR (500 MHz, dmso) δ 7.75 (dd, J = 9.6, 8.7 Hz, 1H), 6.67 (dd, J = 8.8, 1.9 Hz, 1H), 3.78 (ddt, J = 12.9, 3.2, 1.1 Hz, 1H), 3.57 (ddd, J = 13.3, 6.7, 3.8 Hz, 1H), 3.33 – 3.25 (m, 2H), 3.24 – 3.19 (m, 1H), 1.87 (dqd, J = 11.3, 3.8, 1.9 Hz, 1H), 1.72 – 1.61 (m, 1H), 1.54 – 1.46 (m, 1H), 1.43 – 1.34 (m, 1H).

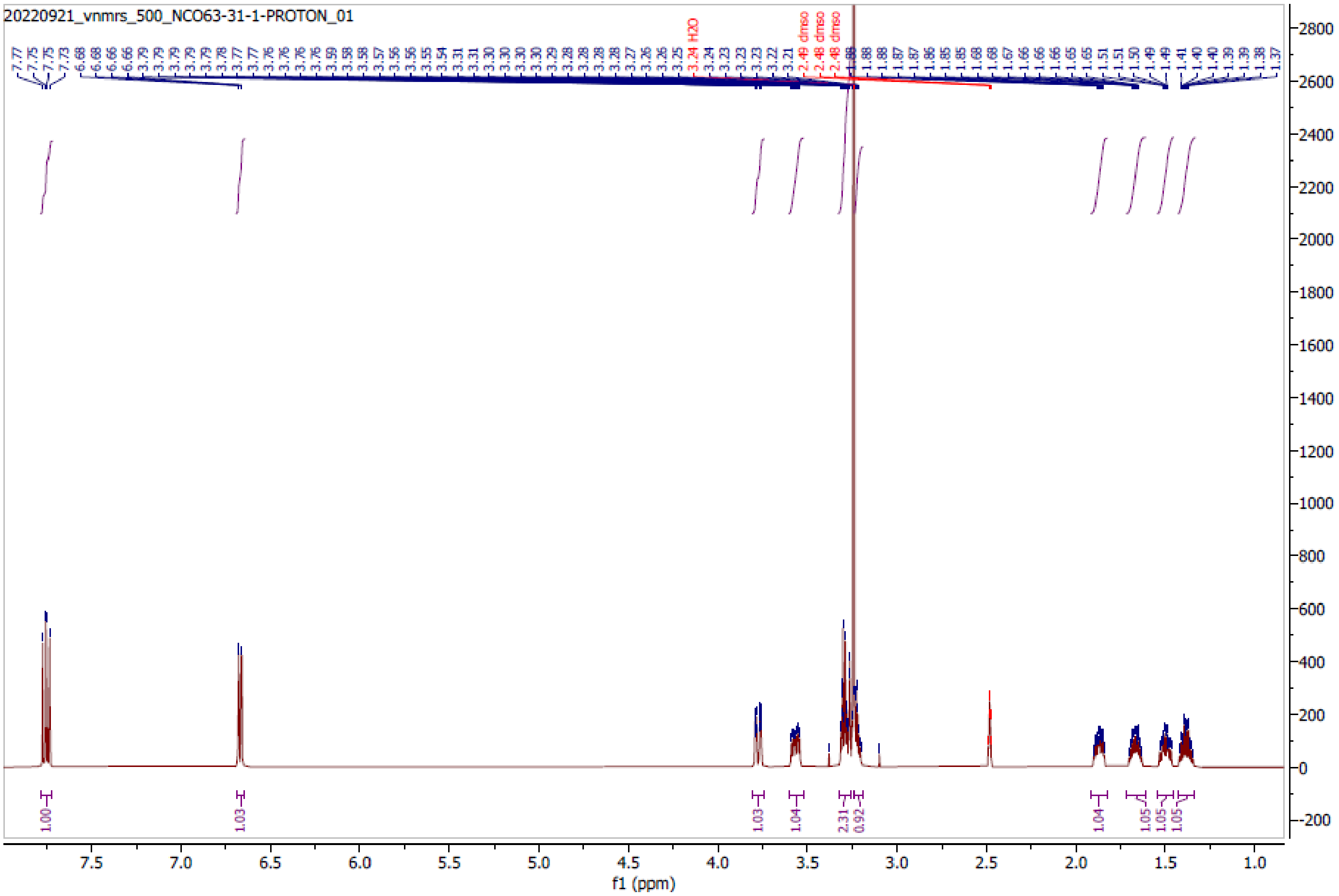

Figure S28. ^{1}H-NMR of 3-Bromo-2-fluoro-6-[(3*S*)-3-methoxypiperidin-1-yl]pyridine (2)

t-Butyl 5-[(t-butyldimethylsilyl)oxy]-2-{2-fluoro-6-[(3*S*)-3-methoxypiperidin-1-yl]pyridin-3-yl}-1*H*-indole-1-carboxylate (3)

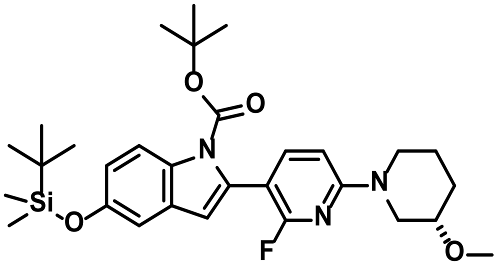

^{13}C NMR (126 MHz, dmso) δ 170.31, 159.27, 157.41, 157.17, 157.04, 153.33, 149.45, 142.00, 141.97, 133.62, 133.58, 130.25, 129.71, 115.39, 113.35, 109.69, 104.99, 102.73, 102.67, 102.64, 102.49, 83.11, 82.96, 74.17, 59.75, 55.56, 48.11, 44.85, 30.96, 29.53, 27.20, 22.07, 21.57, 20.74, 20.04, 14.07, 13.95, 13.92.

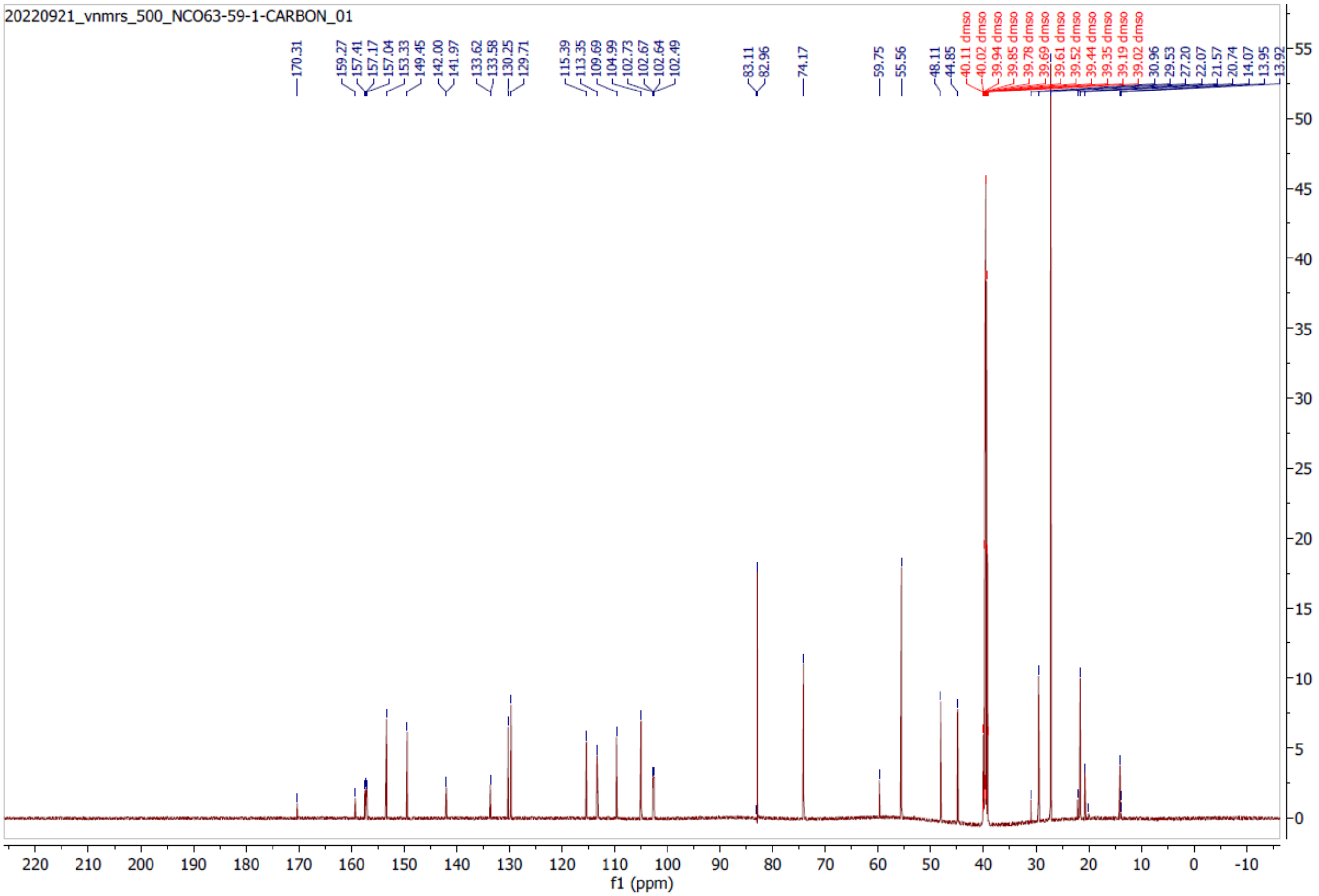

Figure S29. ^{13}C-NMR of t-Butyl 5-[(t-butyldimethylsilyl)oxy]-2-{2-fluoro-6-[(3*S*)-3-methoxypiperidin-1-yl]pyridin-3-yl}-1*H*-indole-1-carboxylate (3)

^{1}H NMR (500 MHz, dmso) δ 7.92 (dt, *J* = 8.9, 0.7 Hz, 1H), 7.66 (dd, *J* = 10.2, 8.3 Hz, 1H), 7.01 (dd, *J* = 2.4, 0.5 Hz, 1H), 6.83 (dd, *J* = 8.9, 2.5 Hz, 1H), 6.76 (dd, *J* = 8.4, 2.1 Hz, 1H), 6.59 (d, *J* = 0.7 Hz, 1H), 3.89 (dd, *J* = 13.1, 3.3 Hz, 1H), 3.67 (ddd, *J* = 13.2, 6.4, 3.8 Hz, 1H), 3.62 – 3.52 (m, 4H), 3.39 – 3.31 (m, 2H), 3.27 (s, 3H), 3.24 (tt, *J* = 7.4, 3.4 Hz, 1H), 1.92 (ddt, *J* = 11.4, 7.4, 3.8 Hz, 1H), 1.78 – 1.65 (m, 5H), 1.53 (dtd, *J* = 12.5, 8.3, 3.9 Hz, 1H), 1.45 – 1.36 (m, 1H), 1.34 (s, 9H), 0.95 (s, 9H).

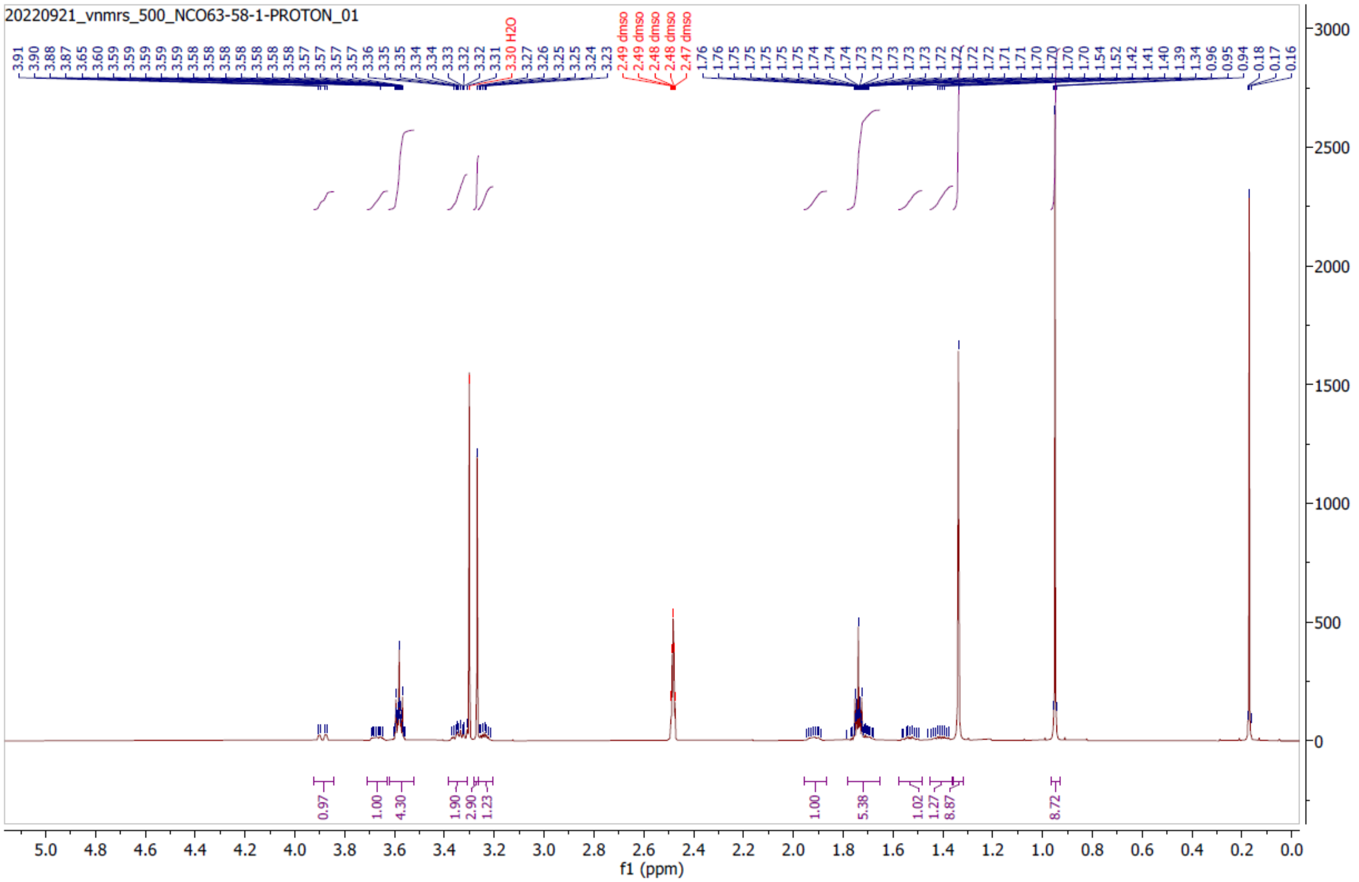

Figure S30. ^{1}H-NMR of t-Butyl 5-[(t-butyldimethylsilyl)oxy]-2-{2-fluoro-6-[(3*S*)-3-methoxypiperidin-1-yl]pyridin-3-yl}-1*H*-indole-1-carboxylate (3)

t-Butyl 2-{2-fluoro-6-[(3*S*)-3-methoxypiperidin-1-yl]pyridin-3-yl}-5-hydroxy-1*H*-indole-1-carboxylate (4)

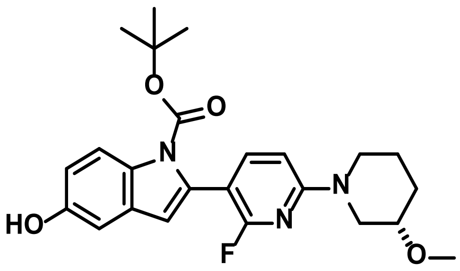

^{13}C NMR (126 MHz, dmso) δ 170.31, 159.27, 157.41, 157.17, 157.04, 153.33, 149.45, 142.00, 141.97, 133.62, 133.58, 130.25, 129.71, 115.39, 113.35, 109.69, 104.99, 102.73, 102.67, 102.64, 102.49, 83.11, 82.96, 74.17, 59.75, 55.56, 48.11, 44.85, 30.96, 29.53, 27.20, 22.07, 21.57, 20.74, 20.04, 14.07, 13.95, 13.92.

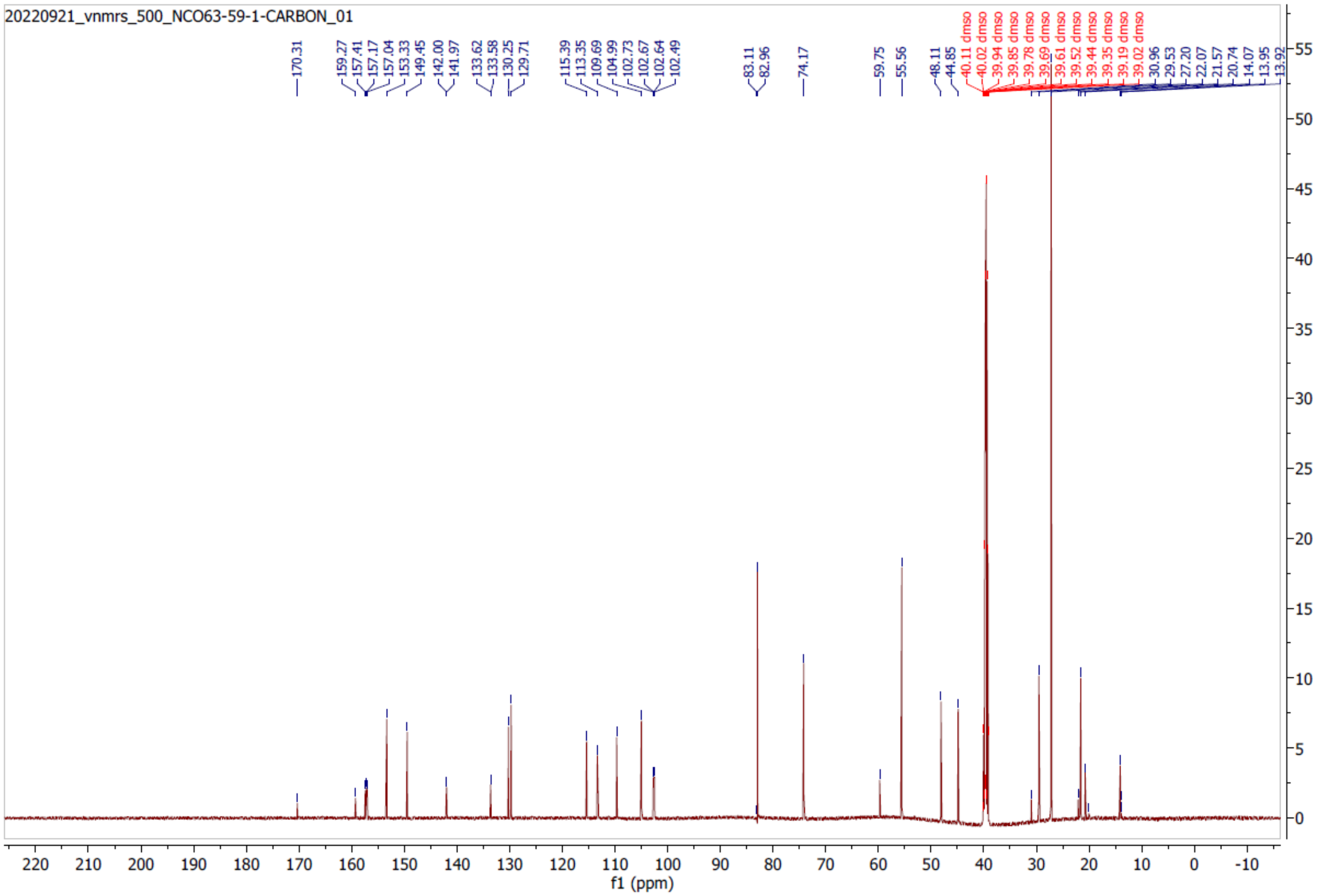

Figure S31. ^{13}C-NMR of t-Butyl 2-{2-fluoro-6-[(3*S*)-3-methoxypiperidin-1-yl]pyridin-3-yl}-5-hydroxy-1*H*-indole-1-carboxylate (4)

^{1}H NMR (500 MHz, dmso) δ 9.18 (s, 1H), 7.87 (dt, *J* = 8.8, 0.7 Hz, 1H), 7.63 (dd, *J* = 10.2, 8.3 Hz, 1H), 6.89 (dd, *J* = 2.5, 0.5 Hz, 1H), 6.77 (dd, *J* = 8.9, 2.5 Hz, 1H), 6.72 (dd, *J* = 8.4, 2.1 Hz, 1H), 6.53 (d, *J* = 0.7 Hz, 1H), 3.88 (dd, *J* = 13.1, 3.3 Hz, 1H), 3.65 (ddd, *J* = 13.2, 6.3, 3.8 Hz, 1H), 3.36 – 3.28 (m, 3H), 3.22 (tt, *J* = 7.4, 3.4 Hz, 1H), 1.94 – 1.85 (m, 1H), 1.69 (dtt, *J* = 13.4, 6.9, 3.5 Hz, 1H), 1.52 (tdd, *J* = 12.5, 7.7, 3.9 Hz, 1H), 1.45 – 1.35 (m, 1H), 1.33 (s, 8H).

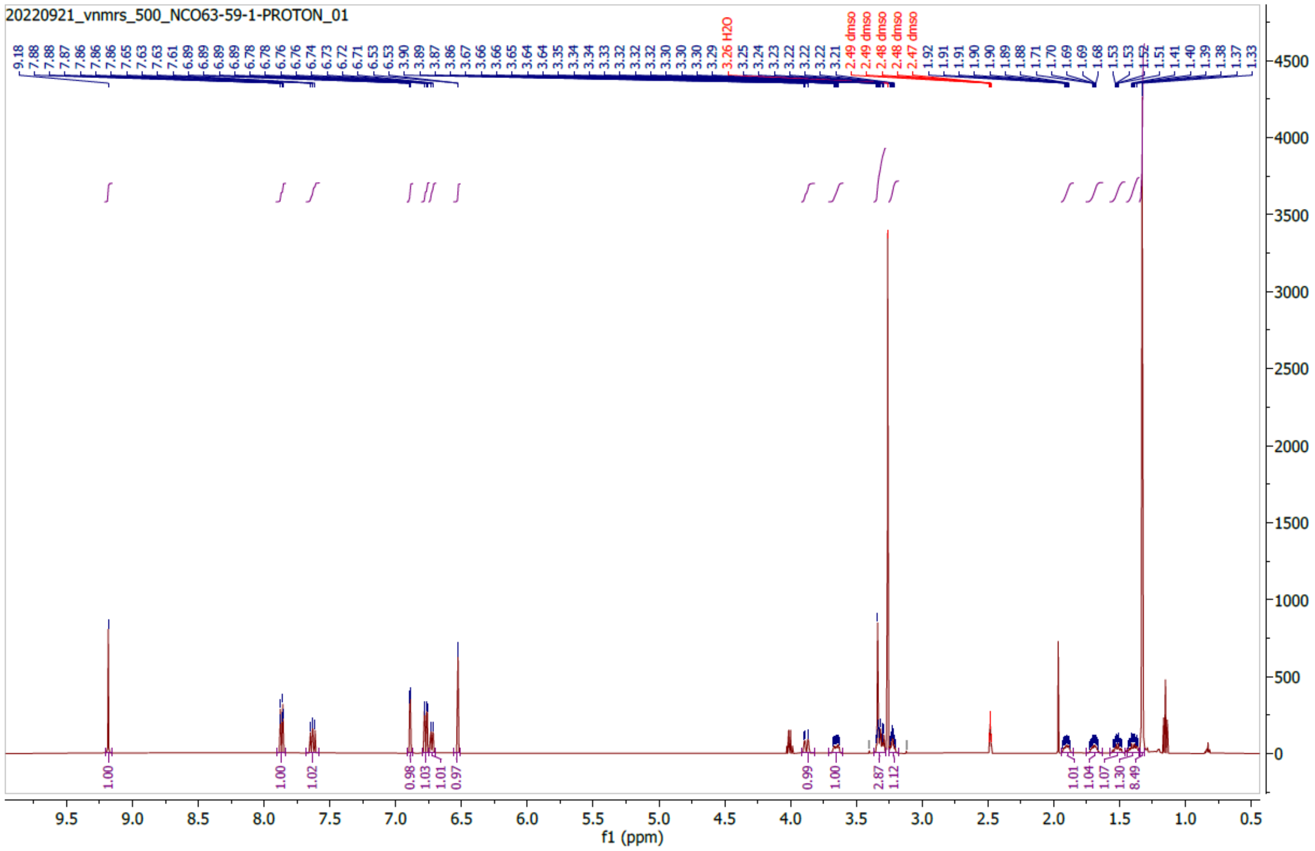

Figure S32. ^{1}H-NMR of t-Butyl 2-{2-fluoro-6-[(3*S*)-3-methoxypiperidin-1-yl]pyridin-3-yl}-5-hydroxy-1*H*-indole-1-carboxylate (4)

2-{2-Fluoro-6-[(3*S*)-3-methoxypiperidin-1-yl]pyridin-3-yl}-1*H*-indol-5-ol (OXD-2314)

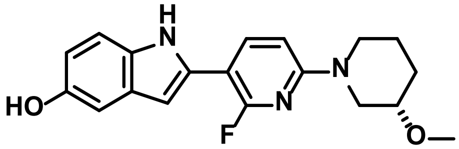

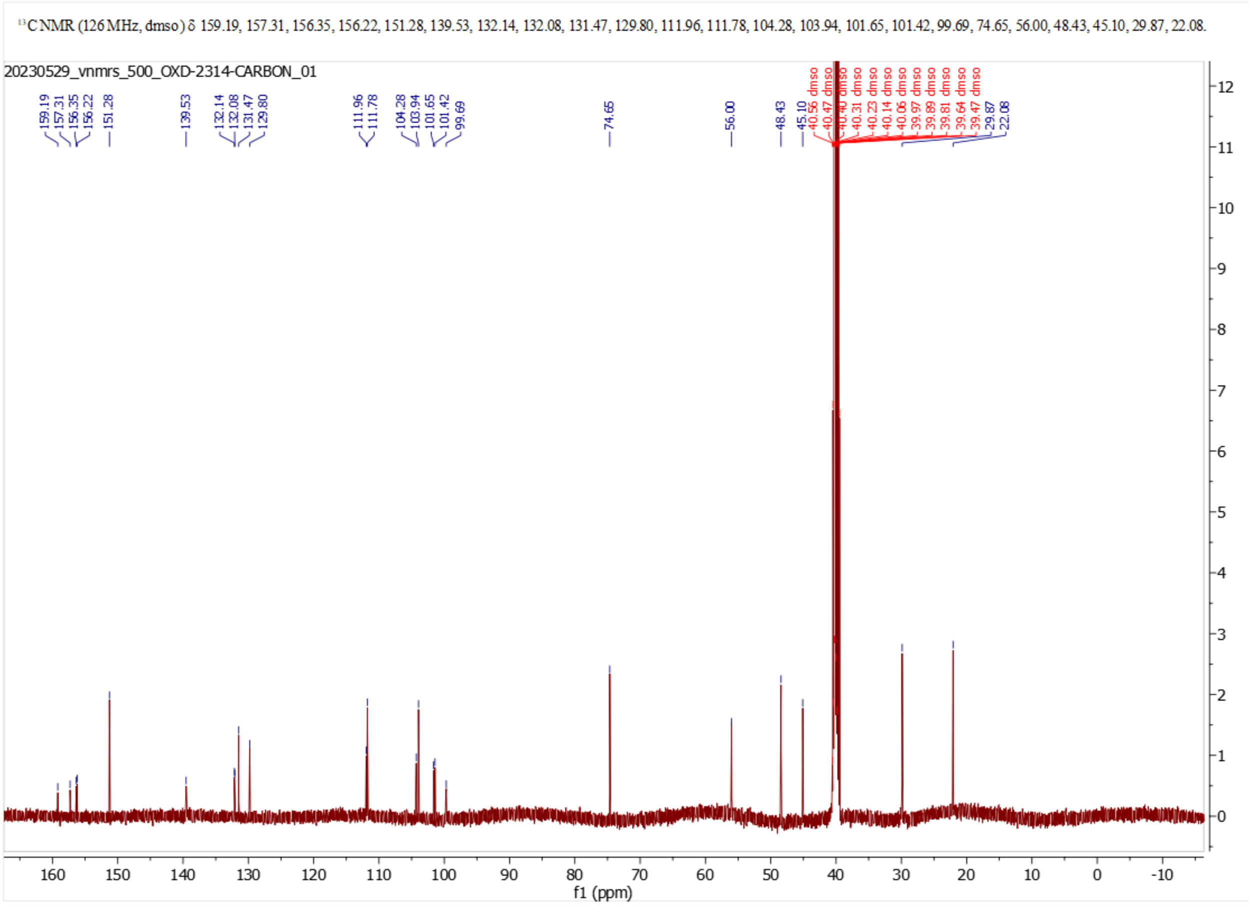

Figure S33. ^{13}C-NMR of 2-{2-Fluoro-6-[(3*S*)-3-methoxypiperidin-1-yl]pyridin-3-yl}-1*H*-indol-5-ol (OXD-2314)

^{1}H NMR (400 MHz, DMSO) δ 11.00 (s, 1H), 8.69 (s, 1H), 8.12 (dd, J = 10.7, 8.6 Hz, 1H), 7.23 (dt, J = 8.6, 0.7 Hz, 1H), 6.91 (dd, J = 8.6, 2.1 Hz, 1H), 6.87 (d, J = 2.3 Hz, 1H), 6.65 (dd, J = 8.6, 2.3 Hz, 1H), 6.60 – 6.53 (m, 1H), 3.97 (d, J = 12.1 Hz, 1H), 3.75 (ddd, J = 13.0, 6.3, 3.7 Hz, 1H), 3.36 (s, 7H), 2.08 – 1.91 (m, 1H), 1.79 (ddt, J = 13.4, 6.8, 3.4 Hz, 1H), 1.67 – 1.42 (m, 2H).

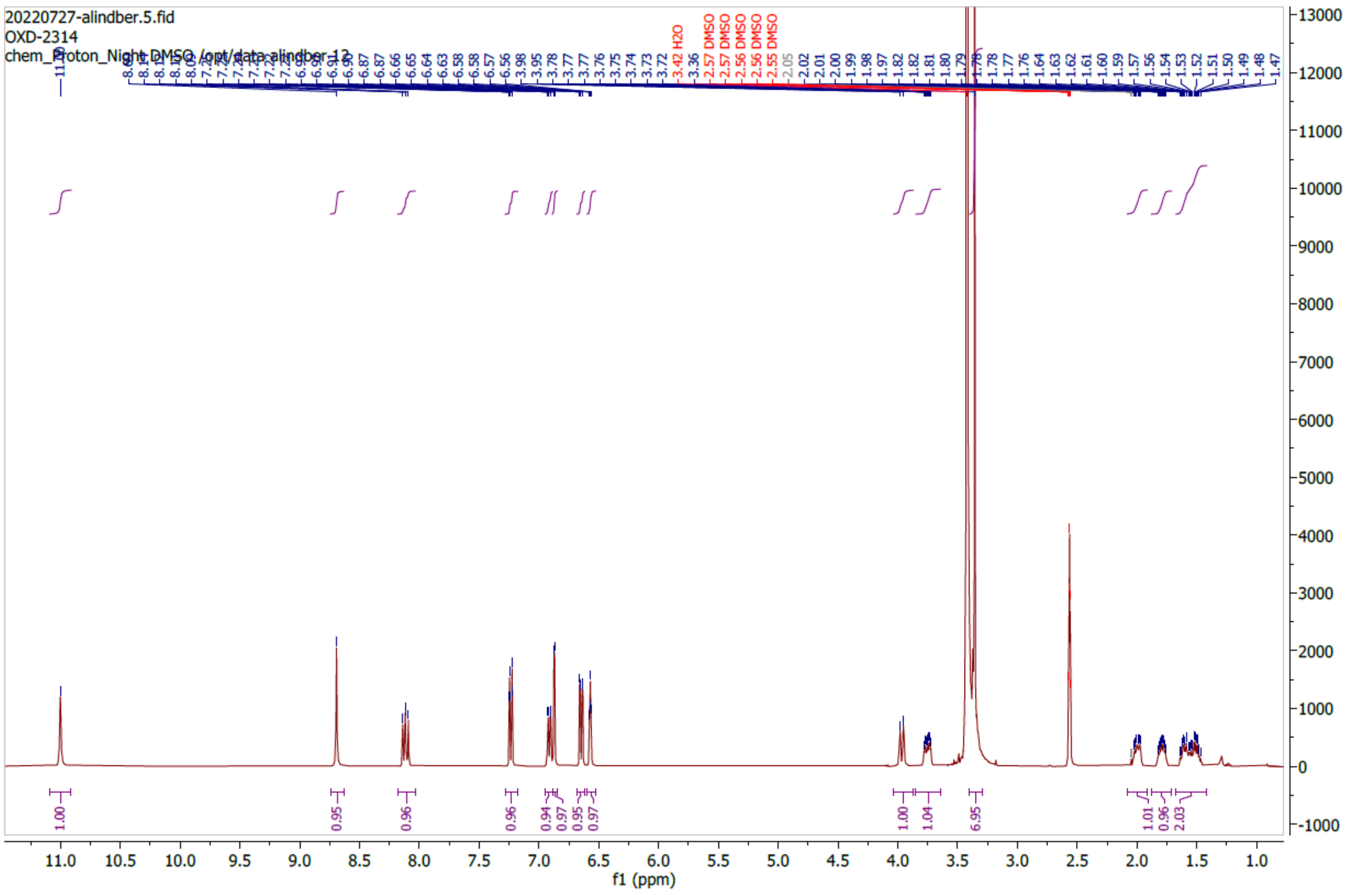

Figure S34. ^{1}H-NMR of 2-{2-Fluoro-6-[(3*S*)-3-methoxypiperidin-1-yl]pyridin-3-yl}-1*H*-indol-5-ol (OXD-2314)

^{19}F NMR (377 MHz, DMSO) δ -67.79, -67.82.

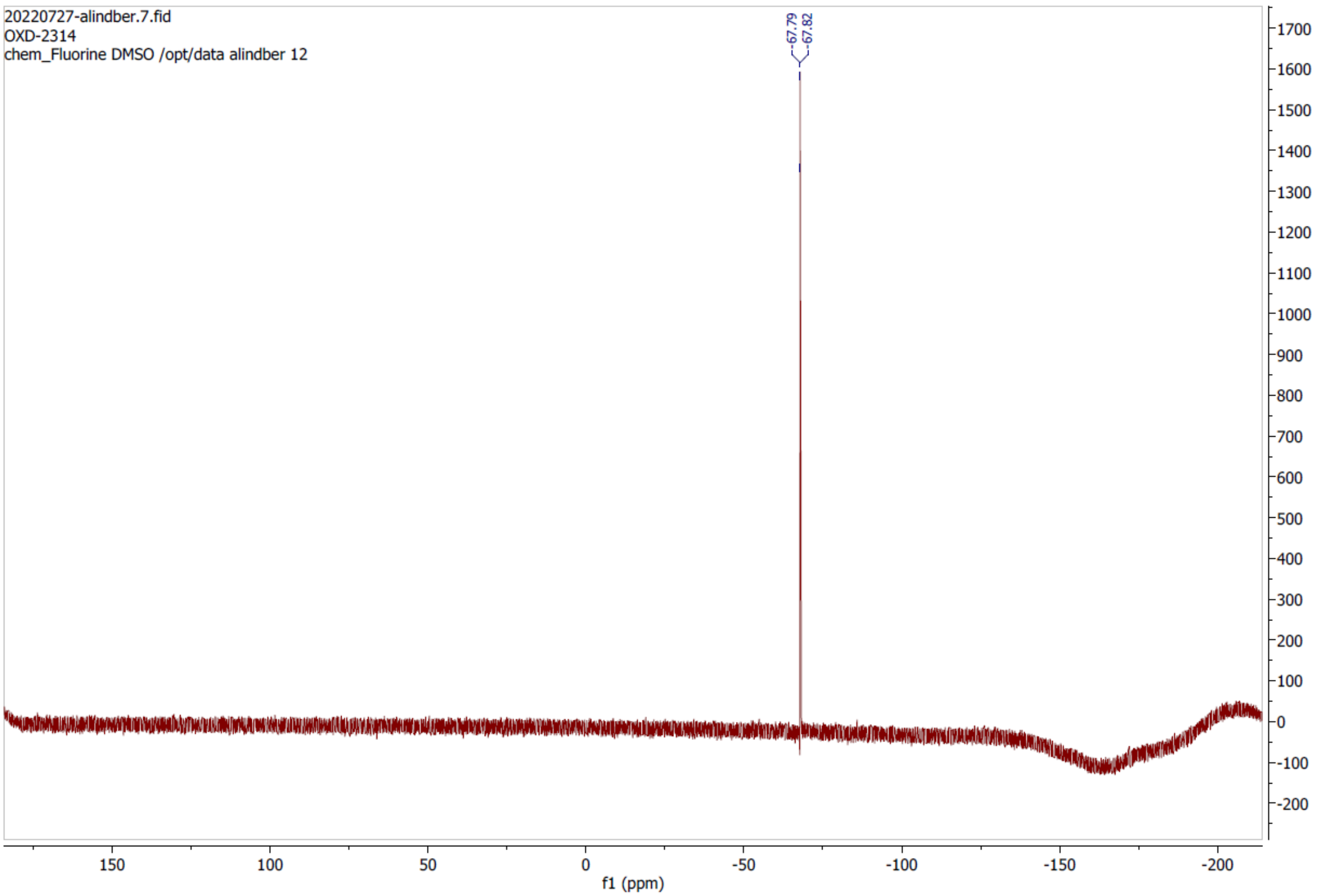

Figure S35. ^{19}F-NMR of 2-{2-Fluoro-6-[(3*S*)-3-methoxypiperidin-1-yl]pyridin-3-yl}-1*H*-indol-5-ol (OXD-2314)

2-[(3*S*)-3-Methoxypiperidin-1-yl]-6-nitropyridine (5)

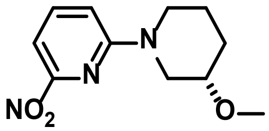

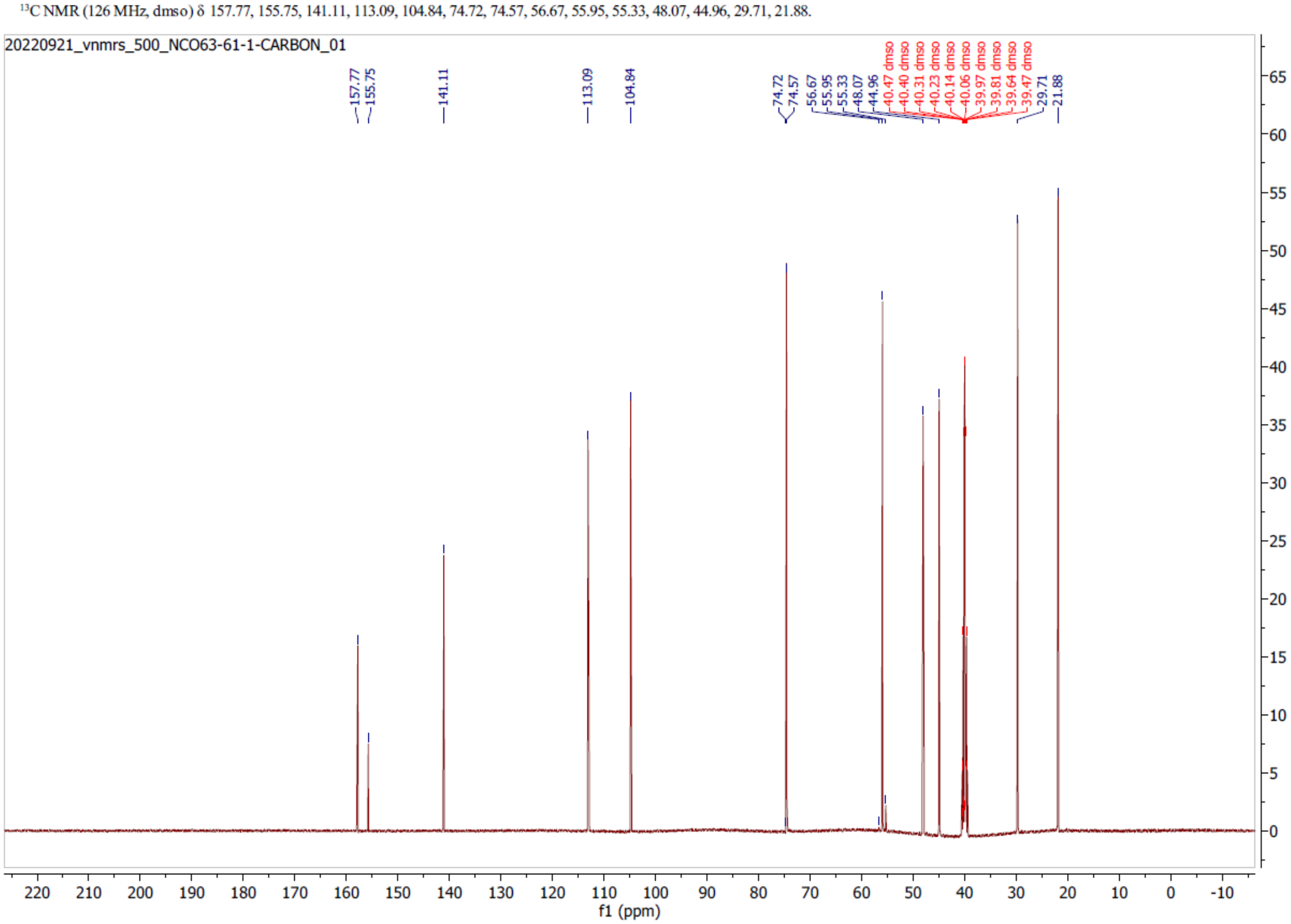

Figure S36. ^{13}C-NMR of 2-[(3*S*)-3-Methoxypiperidin-1-yl]-6-nitropyridine (5)

^{1}H NMR (500 MHz, dmso) δ 7.78 (dd, J = 8.6, 7.5 Hz, 1H), 7.34 (d, J = 7.5 Hz, 1H), 7.25 (d, J = 8.6 Hz, 1H), 3.86 (ddt, J = 13.4, 3.4, 1.0 Hz, 1H), 3.66 (ddd, J = 13.3, 6.9, 3.7 Hz, 1H), 3.50 – 3.41 (m, 2H), 3.30 – 3.26 (m, 1H), 3.25 (s, 3H), 1.92 – 1.84 (m, 1H), 1.70 (dtd, J = 17.0, 7.3, 3.7 Hz, 1H), 1.58 – 1.49 (m, 1H), 1.45 – 1.36 (m, 1H).

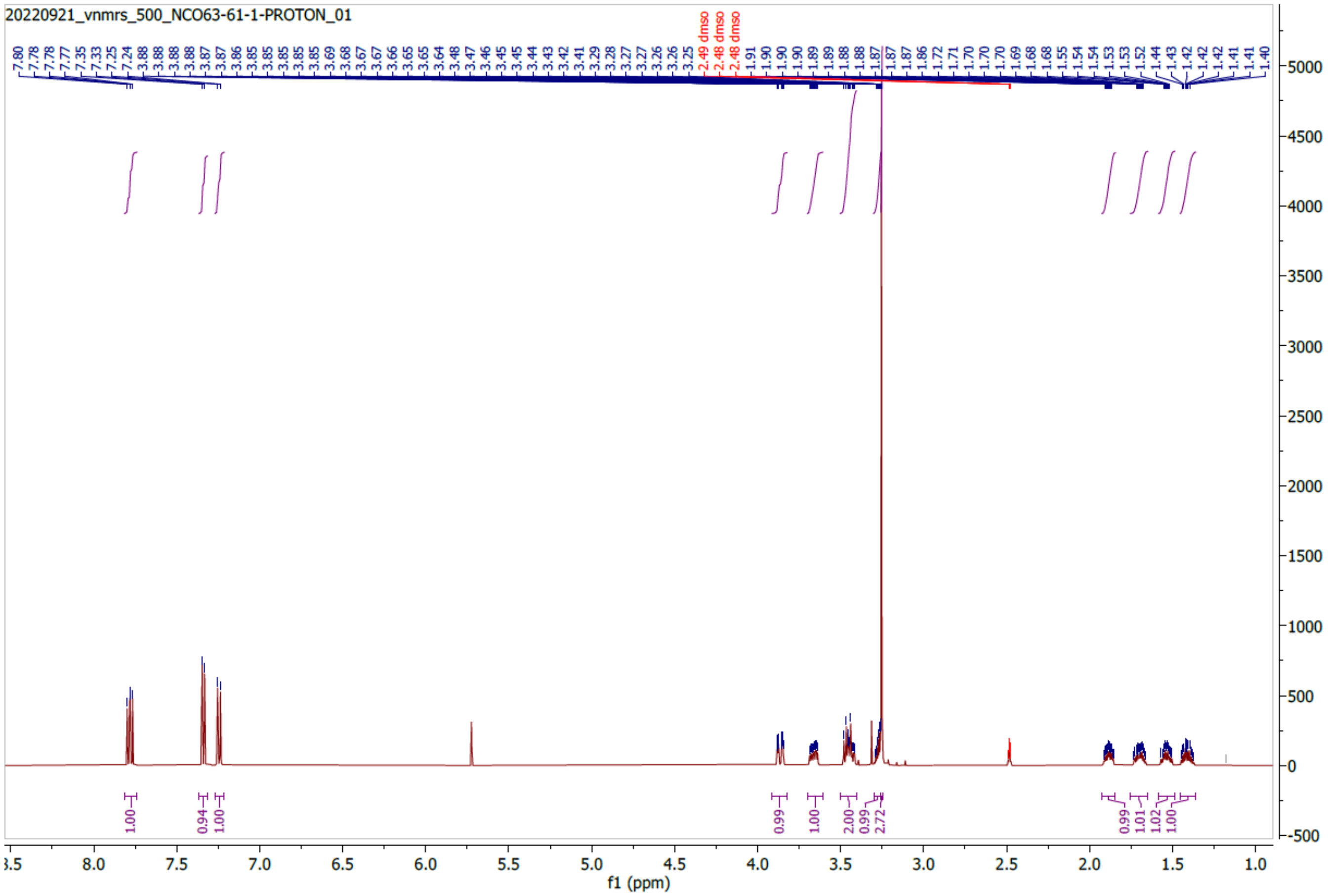

Figure S37. ^{1}H-NMR of 2-[(3*S*)-3-Methoxypiperidin-1-yl]-6-nitropyridine (5)

3-Bromo-6-[(3*S*)-3-methoxypiperidin-1-yl]-2-nitropyridine (6)

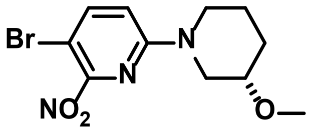

^{13}C NMR (101 MHz, $CDCl_3$-insert) δ 156.28, 144.12, 110.93, 93.14, 77.36, 77.04, 76.73, 74.58, 56.40, 48.46, 45.43, 29.58, 21.80.

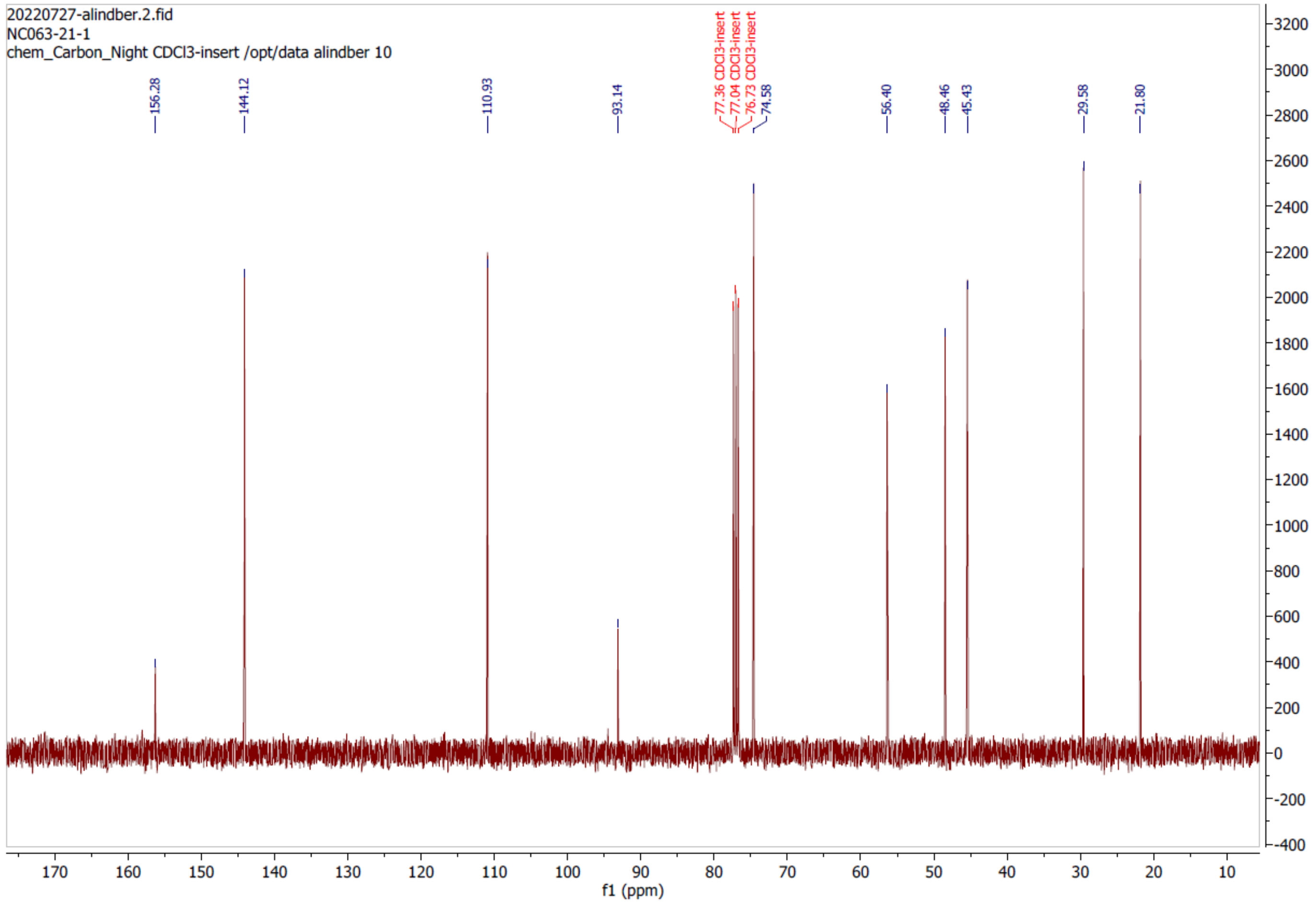

Figure S38. ^{13}C-NMR of 3-Bromo-6-[(3*S*)-3-methoxypiperidin-1-yl]-2-nitropyridine (6)

^1H NMR (400 MHz, $CDCl_3$-insert) δ 7.70 (d, J = 9.0 Hz, 1H), 6.72 (d, J = 9.0 Hz, 1H), 3.85 (ddt, J = 13.2, 3.4, 1.1 Hz, 1H), 3.68 (ddd, J = 13.3, 6.6, 3.8 Hz, 1H), 3.52 – 3.31 (m, 6H), 2.05 – 1.94 (m, 1H), 1.88 (ddp, J = 14.2, 7.3, 3.6 Hz, 1H), 1.69 (ddt, J = 12.8, 8.7, 3.9 Hz, 1H), 1.61 – 1.48 (m, 1H).

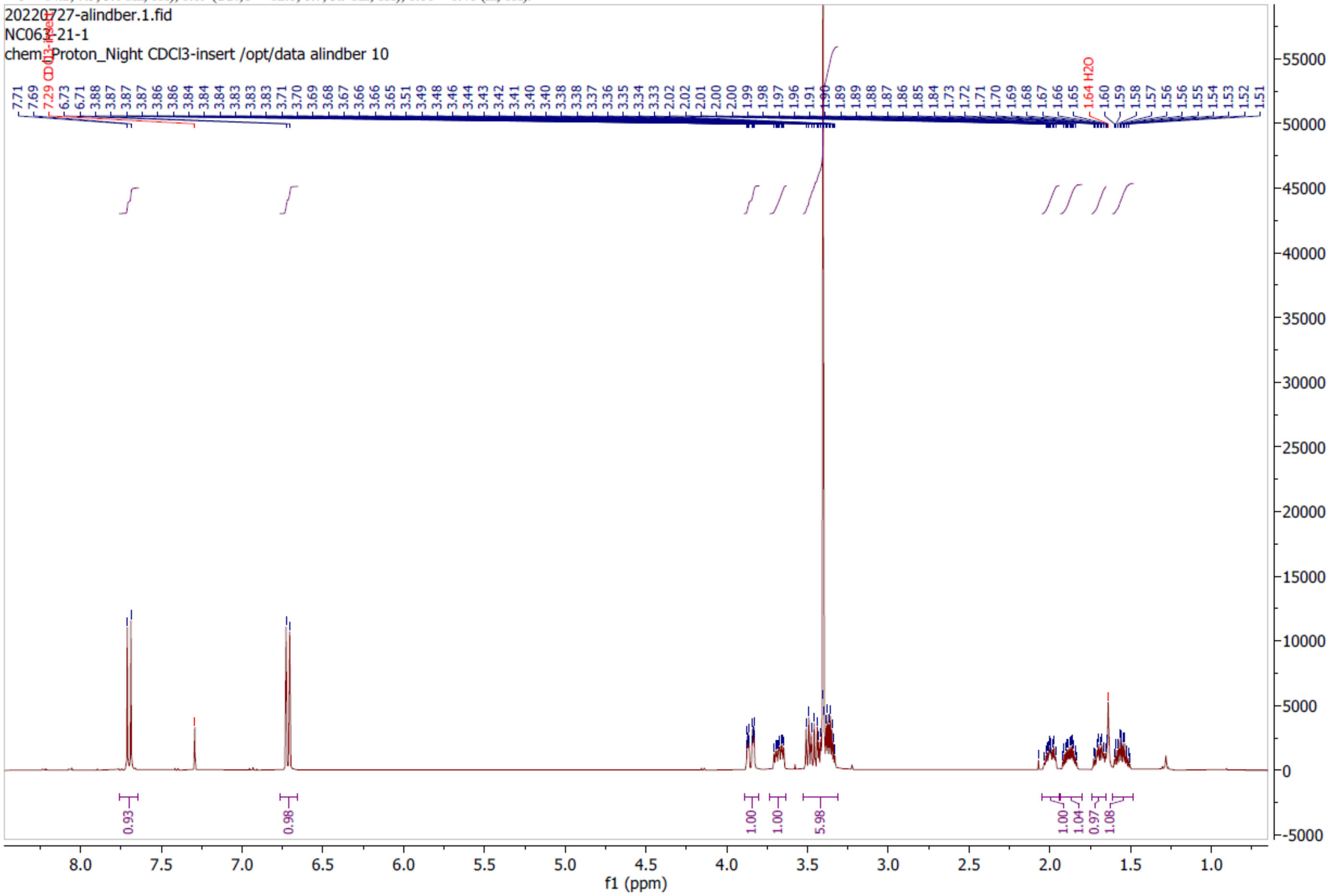

Figure S39. ^{1}H-NMR of 3-Bromo-6-[(3*S*)-3-methoxypiperidin-1-yl]-2-nitropyridine (6)

t-Butyl 5-[(t-butyldimethylsilyl)oxy]-2-{6-[(3*S*)-3-methoxypiperidin-1-yl]-2-nitropyridin-3-yl}-1*H*-indole-1-carboxylate (7)

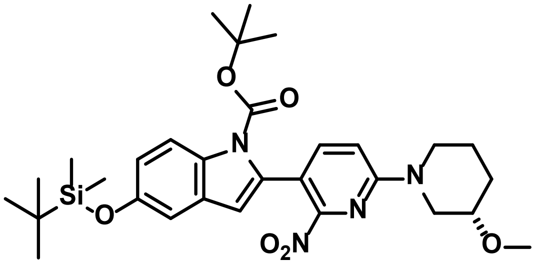

^{13}C NMR (126 MHz, dmso) δ 157.00, 154.26, 151.41, 149.57, 143.55, 134.78, 131.73, 130.17, 118.18, 116.45, 111.10, 110.83, 110.76, 110.46, 83.81, 74.56, 56.04, 48.16, 45.19, 29.72, 27.60, 26.08, 21.82, 18.42, -4.08.

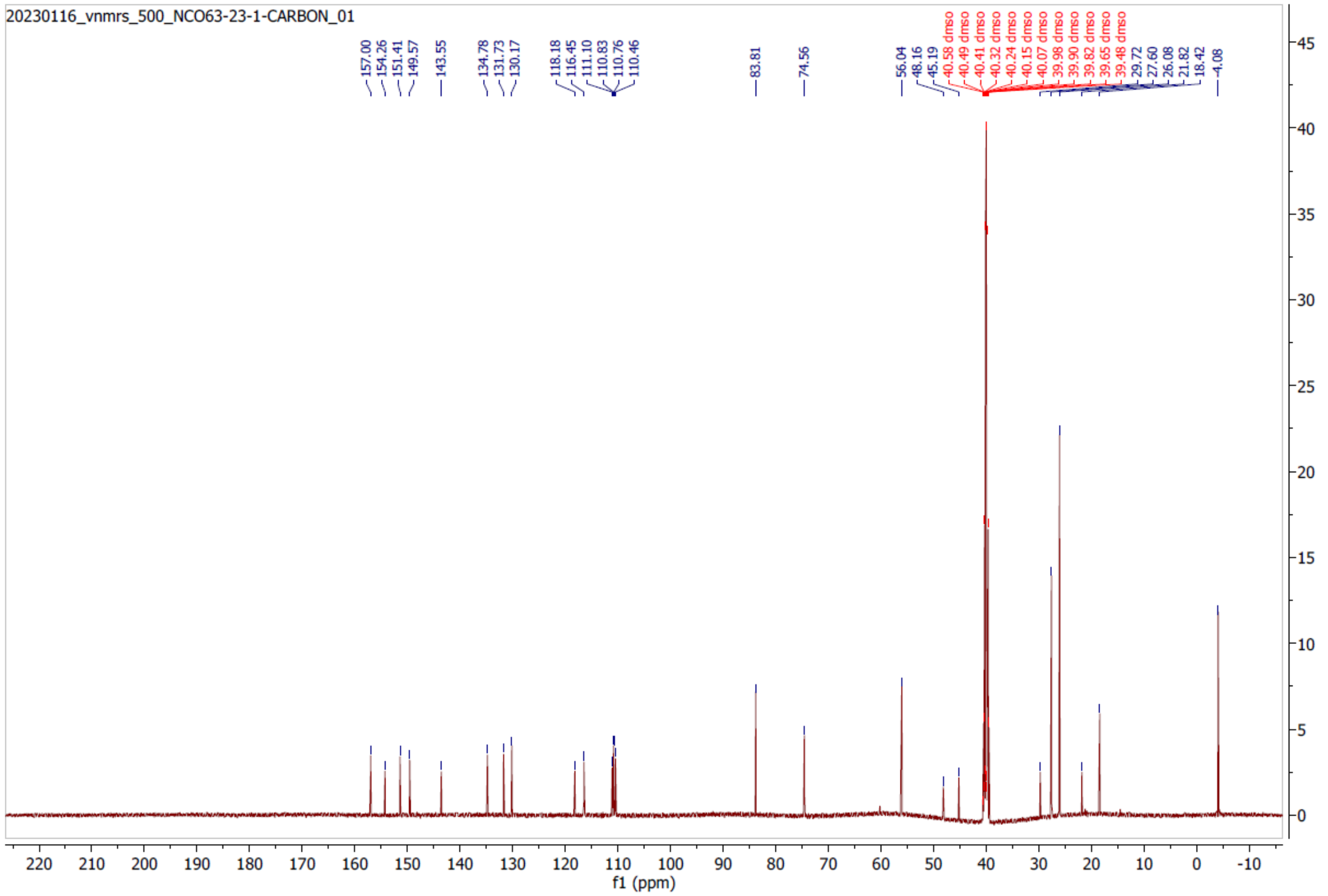

Figure S40. ^{13}C-NMR of t-Butyl 5-[(t-butyldimethylsilyl)oxy]-2-{6-[(3*S*)-3-methoxypiperidin-1-yl]-2-nitropyridin-3-yl}-1*H*-indole-1-carboxylate (7)

^{1}H NMR (500 MHz, dmso) δ 7.97 (dt, J = 9.0, 0.7 Hz, 1H), 7.77 (d, J = 8.7 Hz, 1H), 7.27 (d, J = 8.7 Hz, 1H), 7.04 – 6.99 (m, 1H), 6.85 (dd, J = 9.0, 2.5 Hz, 1H), 6.57 (d, J = 0.7 Hz, 1H), 3.85 (dd, J = 13.5, 3.1 Hz, 1H), 3.66 (ddd, J = 13.0, 7.0, 3.7 Hz, 1H), 3.58 (dd, J = 13.3, 6.9 Hz, 2H), 3.29 (td, J = 6.6, 3.1 Hz, 1H), 3.27 (s, 3H), 1.90 (ddt, J = 12.3, 8.0, 3.9 Hz, 1H), 1.76 – 1.67 (m, 1H), 1.60 (ddd, J = 12.1, 9.8, 5.8 Hz, 1H), 1.48 – 1.37 (m, 1H), 1.28 (s, 12H), 0.95 (s, 12H), 0.18 (s, 6H).

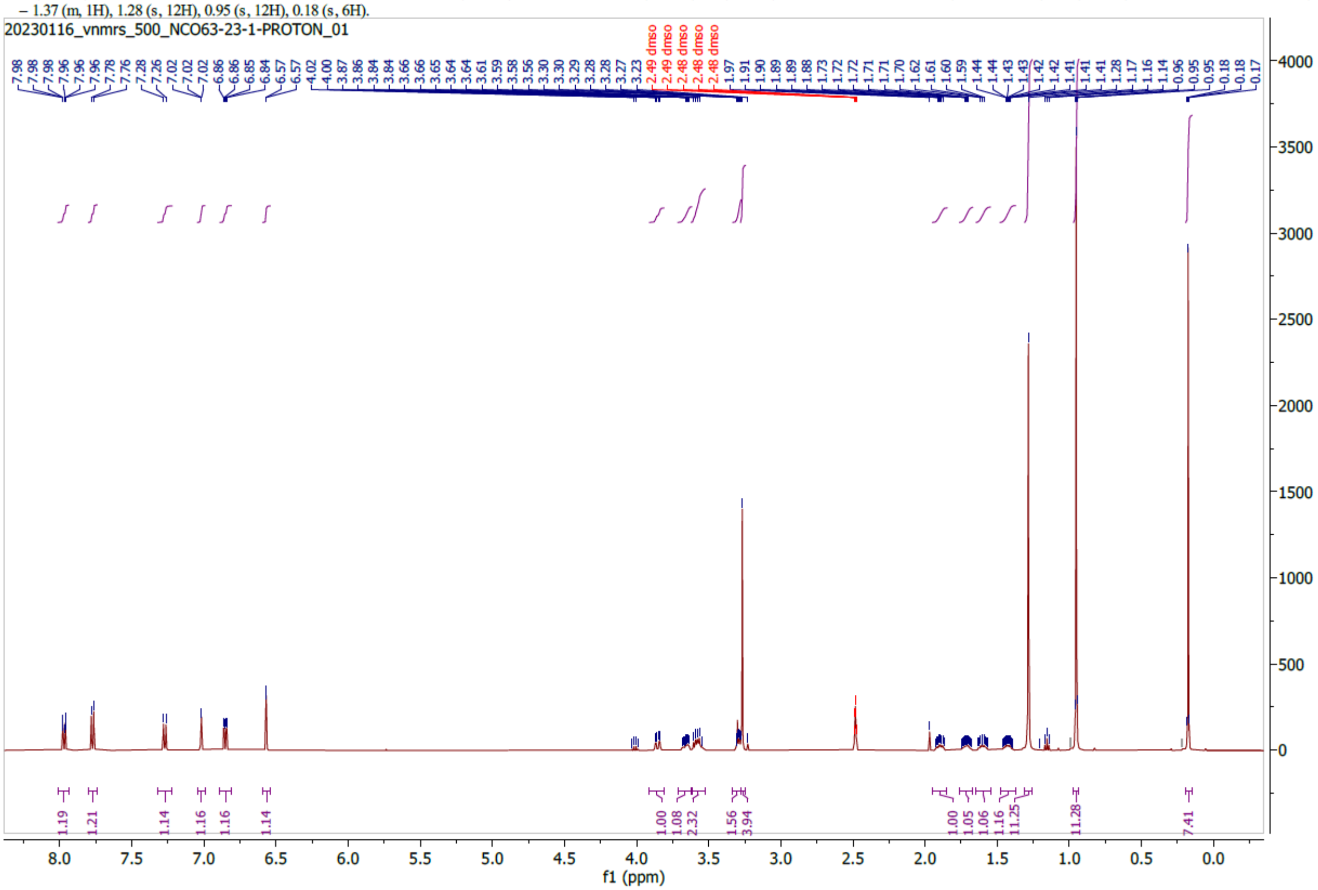

Figure S41. ^{1}H-NMR of t-Butyl 5-[(t-butyldimethylsilyl)oxy]-2-{6-[(3*S*)-3-methoxypiperidin-1-yl]-2-nitropyridin-3-yl}-1*H*-indole-1-carboxylate (7)

t-Butyl 5-hydroxy-2-{6-[(3*S*)-3-methoxypiperidin-1-yl]-2-nitropyridin-3-yl}-1*H*-indole-1-carboxylate (8)

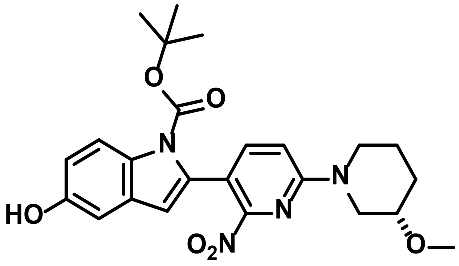

^{13}C NMR (126 MHz, dmso) δ 170.78, 156.96, 154.31, 153.84, 149.63, 143.54, 134.35, 130.38, 130.22, 116.36, 114.06, 111.06, 110.92, 110.47, 105.63, 83.54, 74.56, 60.20, 56.04, 55.36, 48.17, 45.20, 29.72, 27.63, 21.82, 21.21, 14.53.

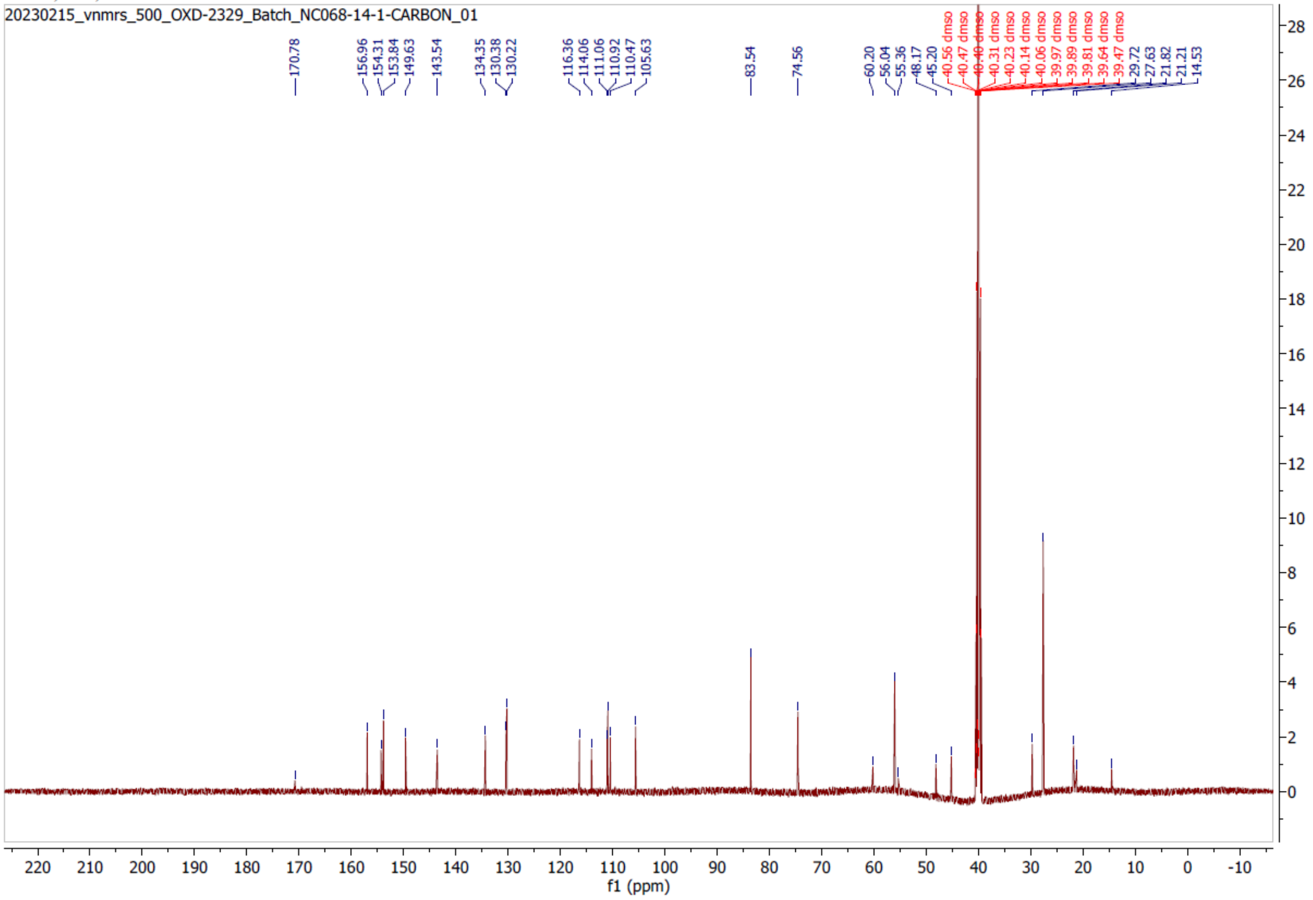

Figure S42. ^{13}C-NMR of t-Butyl 5-hydroxy-2-{6-[(3*S*)-3-methoxypiperidin-1-yl]-2-nitropyridin-3-yl}-1*H*-indole-1-carboxylate (8)

^{1}H NMR (500 MHz, dmso) δ 9.22 (s, 1H), 7.89 (dt, J = 8.9, 0.7 Hz, 1H), 7.76 (d, J = 8.7 Hz, 1H), 7.26 (d, J = 8.8 Hz, 1H), 6.89 (dd, J = 2.5, 0.5 Hz, 1H), 6.78 (dd, J = 8.9, 2.5 Hz, 1H), 6.51 (d, J = 0.7 Hz, 1H), 3.85 (dd, J = 13.5, 3.1 Hz, 1H), 3.66 (ddd, J = 13.2, 7.0, 3.6 Hz, 1H), 3.57 (dt, J = 15.3, 7.7 Hz, 2H), 3.31 (s, 3H), 3.29 (dd, J = 6.9, 3.5 Hz, 1H), 1.90 (ddt, J = 12.2, 7.9, 4.2 Hz, 1H), 1.77 – 1.66 (m, 1H), 1.60 (ddd, J = 12.1, 9.9, 5.8 Hz, 1H), 1.49 – 1.37 (m, 1H), 1.28 (s, 9H).

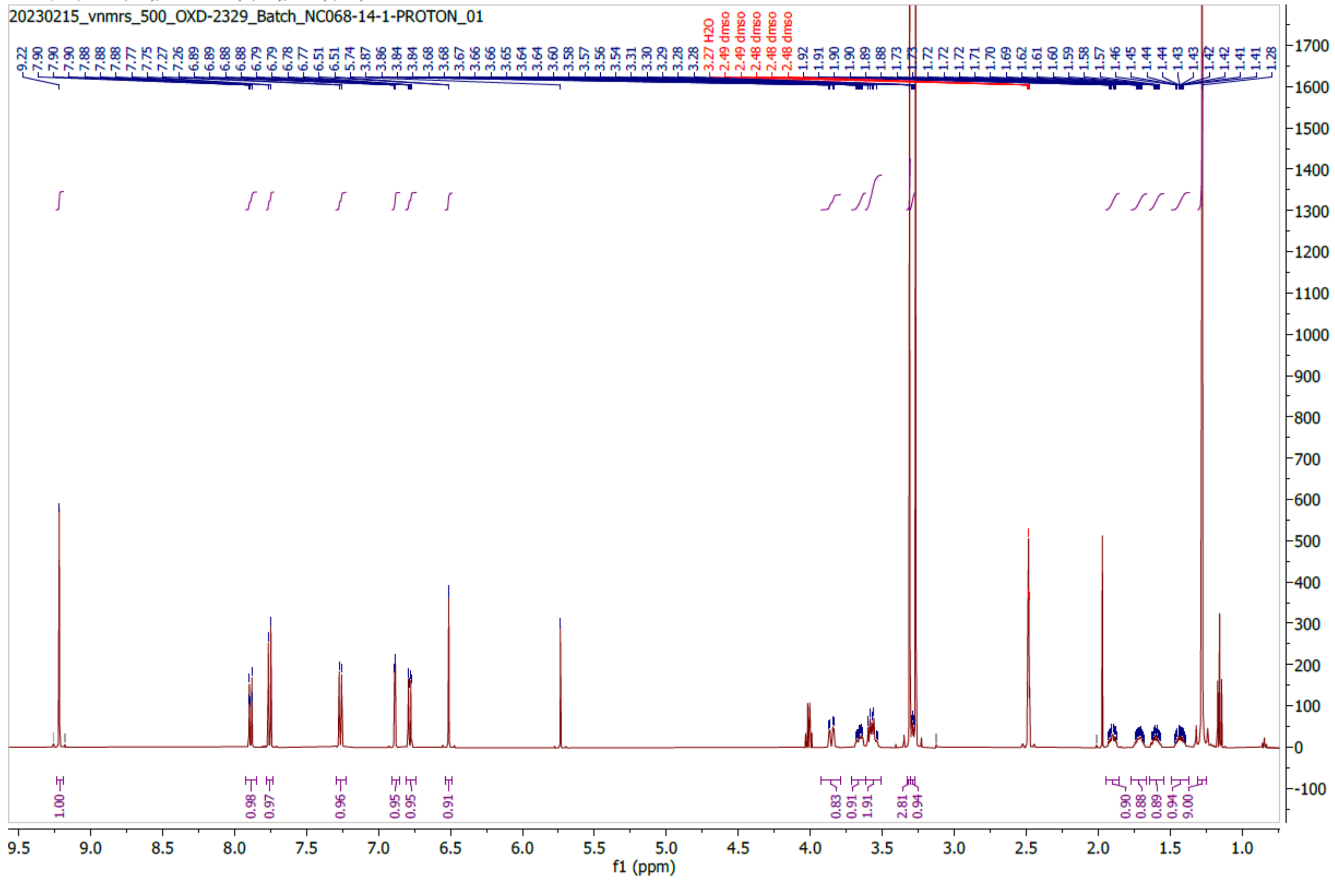

Figure S43. ^{1}H-NMR of t-Butyl 5-hydroxy-2-{6-[(3*S*)-3-methoxypiperidin-1-yl]-2-nitropyridin-3-yl}-1*H*-indole-1-carboxylate (8)

Supplementary References

1 Lindberg, A. *et al.* Radiosynthesis, In Vitro and In Vivo Evaluation of [^{18}F]CBD-2115 as a First-in-Class Radiotracer for Imaging 4R-Tauopathies. *ACS Chemical Neuroscience* **12**, 596-602 (2021). https://doi.org/10.1021/acschemneuro.0c00801
