# Supplementary material for: Ligand-based design of [18F]OXD-2314 for PET imaging in non-Alzheimer’s disease tauopathies
Source: Nat Commun. 2024 Jun 14;15:5109. doi: 10.1038/s41467-024-49258-1 (PMC11178805; doi:10.1038/s41467-024-49258-1)
Supplement: Supplementary file 3 — Reporting Summary [file 41467_2024_49258_MOESM3_ESM.pdf]

nature portfolio

Corresponding author(s): Samuel Svensson, Chester Mathis, Neil Vasdev

Last updated by author(s): May 8, 2024

Reporting Summary

Nature Portfolio wishes to improve the reproducibility of the work that we publish. This form provides structure for consistency and transparency in reporting. For further information on Nature Portfolio policies, see our Editorial Policies and the Editorial Policy Checklist.

Statistics

For all statistical analyses, confirm that the following items are present in the figure legend, table legend, main text, or Methods section.

n/a	Confirmed	
[ ]	[x]	The exact sample size (*n*) for each experimental group/condition, given as a discrete number and unit of measurement
[ ]	[x]	A statement on whether measurements were taken from distinct samples or whether the same sample was measured repeatedly
[x]	[ ]	The statistical test(s) used AND whether they are one- or two-sided<br>*Only common tests should be described solely by name; describe more complex techniques in the Methods section.*
[ ]	[x]	A description of all covariates tested
[x]	[ ]	A description of any assumptions or corrections, such as tests of normality and adjustment for multiple comparisons
[ ]	[x]	A full description of the statistical parameters including central tendency (e.g. means) or other basic estimates (e.g. regression coefficient) AND variation (e.g. standard deviation) or associated estimates of uncertainty (e.g. confidence intervals)
[x]	[ ]	For null hypothesis testing, the test statistic (e.g. *F*, *t*, *r*) with confidence intervals, effect sizes, degrees of freedom and *P* value noted<br>*Give P values as exact values whenever suitable.*
[x]	[ ]	For Bayesian analysis, information on the choice of priors and Markov chain Monte Carlo settings
[x]	[ ]	For hierarchical and complex designs, identification of the appropriate level for tests and full reporting of outcomes
[x]	[ ]	Estimates of effect sizes (e.g. Cohen's *d*, Pearson's *r*), indicating how they were calculated

Our web collection on statistics for biologists contains articles on many of the points above.

Software and code

Policy information about availability of computer code

Data collection	Not applicable - no code used
Data analysis	Not applicable - no code used

For manuscripts utilizing custom algorithms or software that are central to the research but not yet described in published literature, software must be made available to editors and reviewers. We strongly encourage code deposition in a community repository (e.g. GitHub). See the Nature Portfolio guidelines for submitting code & software for further information.

Data

Policy information about availability of data

All manuscripts must include a data availability statement. This statement should provide the following information, where applicable:

- Accession codes, unique identifiers, or web links for publicly available datasets
- A description of any restrictions on data availability
- For clinical datasets or third party data, please ensure that the statement adheres to our policy

All data is available from corresponding authors upon request

Research involving human participants, their data, or biological material

Policy information about studies with human participants or human data. See also policy information about sex, gender (identity/presentation), and sexual orientation and race, ethnicity and racism.

Reporting on sex and gender	Sex difference in pharmacokinetics of [18F]OXD-2314 were studied in rats and non-human primates.<br><br>Sex and age information of human tissue samples were collected and are reported in Table S1
Reporting on race, ethnicity, or other socially relevant groupings	Not applicable - none such used in nature of study.
Population characteristics	Sex and age information of human tissue samples were collected and are reported in Table S1
Recruitment	Not applicable - no human participants used
Ethics oversight	Postmortem human brain tissues were provided by brain banks at the University of California, San Francisco (UCSF) and Banner/Sun Health AZ following informed consent of the donors and utilized at the University of Pittsburgh under the approval of the Committee for Oversight of Research and Clinical Training Involving Decedents and Centre for Addiction and Mental Health Research Ethics Board<br>Rat imaging studies were carried out in accordance with the guidelines put forth by the institutional animal care and use committee at the Centre for Addiction and Mental Health.<br>NHP imaging studies were carried out in accordance with the guidelines set forth by the institutional animal care and use committee at the University of Pittsburgh.

Note that full information on the approval of the study protocol must also be provided in the manuscript.

Field-specific reporting

Please select the one below that is the best fit for your research. If you are not sure, read the appropriate sections before making your selection.

☒ Life sciences ☐ Behavioural & social sciences ☐ Ecological, evolutionary & environmental sciences

For a reference copy of the document with all sections, see nature.com/documents/nr-reporting-summary-flat.pdf

Life sciences study design

All studies must disclose on these points even when the disclosure is negative.

Sample size	Samples sizes for in vitro assays were determined based on tissue availability and in vivo studies were carried out with the minimum number of animals to maintain ethical integrity.
Data exclusions	Not applicable - none excluded
Replication	Studies were performed at multiple sites
Randomization	Not applicable - not relevant to nature of studies
Blinding	Not applicable - not relevant to nature of studies

Reporting for specific materials, systems and methods

We require information from authors about some types of materials, experimental systems and methods used in many studies. Here, indicate whether each material, system or method listed is relevant to your study. If you are not sure if a list item applies to your research, read the appropriate section before selecting a response.

Materials & experimental systems

n/a	Involved in the study	
☐	☒	Antibodies
☒	☐	Eukaryotic cell lines
☒	☐	Palaeontology and archaeology
☐	☒	Animals and other organisms
☒	☐	Clinical data
☒	☐	Dual use research of concern
☒	☐	Plants

Methods

n/a	Involved in the study	
☒	☐	ChIP-seq
☒	☐	Flow cytometry
☒	☐	MRI-based neuroimaging

Antibodies

Antibodies used

Invitrogen Phospho-Tau (Ser202, Thr205) Monoclonal Antibody (AT8) Cat# MN1020, OmniMap DAB Goat- anti-Ms (Cat# 760-150)

Validation

Invitrogen Advanced Verification:
Advanced verification is additional testing that verifies that an antibody will bind to the correct target. We consider two key factors when selecting the appropriate advanced verification testing method(s):
The nature of the target—this includes considering relevant target properties such as its biological function, sub-cellular localization, post-translational modifications, gene essentiality, and the rate of turnover.
The nature of the sample type—the sample type can influence epitope exposure and the reactivity of an antibody in a specific application.

A Peroxidase (HRP)-conjugated anti-mouseantibody, supplied by Roche, cited in 176 publications. Applications used include IHC, IHC-IF, FC/FACS, IF, and 1 other. OmniMap anti-Rb HRP, OmniMap anti-Ms HRP, OmniMap anti-Rt HRP, and OmniMap anti-Gt HRP are biotin-free conjugates based on proprietary multimer technology. They consist of a robust chemistry that provides clean background in combination with enhanced specificity and sensitivity, which increases the signal-to-noise ratio. They are designed to be used in conjunction with the DISCOVERY series of instruments and Ventana Medical Systems' ancillary reagents for optimal performance.

Animals and other research organisms

Policy information about studies involving animals; ARRIVE guidelines recommended for reporting animal research, and Sex and Gender in Research

Laboratory animals

8 healthy adult rats (Sprague Dawley, 384 ± 56 g; 4/4 M/F) underwent PET imaging
Five adult Sprague-Dawley rats (3F/2M, 378 ± 51 g) were used in [18F]OXD-2314 radiometabolite analysis.
Dynamic [18F]OXD-2314 PET imaging was performed in a male macaca mulatta (13 kg) and male (25.7 kg) and female (14.2 kg) baboons

Wild animals

Not applicable - none used

Reporting on sex

See above

Field-collected samples

not applicable - none used

Ethics oversight

Rat imaging studies were carried out in accordance with the guidelines put forth by the institutional animal care and use committee at the Centre for Addiction and Mental Health.
NHP imaging studies were carried out in accordance with the guidelines set forth by the institutional animal care and use committee at the University of Pittsburgh.

Note that full information on the approval of the study protocol must also be provided in the manuscript.

Plants

Seed stocks	not applicable - none used
Novel plant genotypes	not applicable - none used
Authentication	not applicable - none used
